# The Floor‐Ceiling‐Chip, or 2 × 2D = Pseudo‐3D—Approaching 3D Cell Morphology and Organization between Two Opposing 2D Substrates with Cell‐Adhesive Protein Micropatterns

**DOI:** 10.1002/adhm.202503591

**Published:** 2026-03-17

**Authors:** Urandelger Tuvshindorj, Francesca Giacomini, Esra Güben Kaçmaz, Tim ten Brink, Lorenzo Moroni, Zeinab Tahmasebi Birgani, Clemens van Blitterswijk, Pamela Habibović, Stefan Giselbrecht, Jan de Boer, Roman Truckenmüller

**Affiliations:** ^1^ MERLN Institute for Technology‐Inspired Regenerative Medicine Maastricht University Maastricht The Netherlands; ^2^ Department of Biomedical Engineering and Institute for Complex Molecular Systems Eindhoven University of Technology Eindhoven The Netherlands

**Keywords:** 3D cell culture, cell morphology, ion‐track etched membranes, mechanotransduction, protein micropatterning

## Abstract

In this proof‐of‐concept study, we develop a novel 3D cell patterning and culture platform. The “Floor‐Ceiling‐Chip” (FC‐Chip) simply consists of two opposing 2D substrates in the form of ion track‐etched membranes, creating a pseudo‐3D microenvironment for the cells between them. This allows the stimulation of both the dorsal and ventral sides of cells, thereby also eliminating the artificial polarization, for example, of stromal cells, observed in standard culture dishes and inserts. By providing the membranes with micropatterned cell‐adhesive islands of varying geometries and sizes, the FC‐Chip enables control over cell shape and alignment in a 3D environment. Analysis of fluorescence microscopic images reveals distinct cellular and nuclear morphology, along with perinuclear actin organization, in the on‐chip cultures compared to cultures on traditional 2D substrates. Cells in the FC‐Chip exhibit fewer focal adhesions, lower expression of lamin A/C, and less nuclear localization of the yes‐associated protein 1. The chip demonstrates compatibility with standard biochemical assays and supports long‐term cultures up to 10 days, expanding its potential applications. Overall, the early version of the FC‐Chip presented here confirms the feasibility of a straightforward, accessible, and versatile future culture platform for the manipulation and modeling of cell morphology and organization in 3D.

## Graphical abstract

Here, we present a novel 3D cell patterning and culture platform. The “Floor‐Ceiling‐Chip” (FC‐Chip) consists of two opposing track‐etched membranes, creating a pseudo‐3D microenvironment for the cells in between. By providing the membranes with micropatterned cell‐adhesive islands of varying geometries and sizes, the FC‐Chip enables control over cell shape and alignment in 3D.

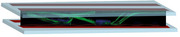

**Abbreviations**
2D: Two‐dimensional
3D: Three‐dimensional
ALP: Alkaline phosphatase
AU: Arbitrary units
BSA: Bovine serum albumin
DM: (Osteogenic) differentiation medium
DUV: Deep UV
ECM: Extracellular matrix
EthD: Ethidium homodimer
F‐actin: Filamentous actin
FC‐Chip: Floor‐Ceiling‐Chip
G‐actin: Globular actin
GM: Growth medium
hMSC: Human mesenchymal stem/stromal cell
PBS: Phosphate‐buffered saline
PC: Polycarbonate
PLL‐g‐PEG: Poly(L‐lysine)‐graft‐poly(ethylene glycol)
RT: Room temperature
SEM: Scanning electron microscopy
YAP: Yes‐associated protein

## 1 Introduction

In vivo, cells often reside in a 3D manner within a complex extracellular matrix (*ECM*). This 3D matrix provides essential biochemical and biophysical cues for proper cell function, differentiation, migration, and tissue morphogenesis [1, 2, 3]. However, conventional cell culture systems such as culture dishes and inserts rely on a reductionist approach where cells are grown with one side on a planar and smooth polymer or glass surface [4]. Such 2D in vitro models fail to recapitulate the intricate 3D microenvironment found in native tissues. These oversimplified approaches also result in an artificial polarization of cells, which can alter cell behavior and function [5, 6, 7], limiting the physiological relevance of experimental findings from these systems or models.

In addition to these 2D cell culture models, 3D in vitro models, such as spheroids [8], organoids [9, 10], and tissue‐engineered constructs, have been developed that better mimic the complex structure and cellular interactions found in vivo [11, 12, 13]. These models have demonstrated improved preservation of cellular phenotypes and functions compared to traditional 2D cultures. For example, human umbilical cord mesenchymal stromal cells (hUC‐MSCs) cultured in spheroids express higher levels of stem cell markers and display stronger multipotency relative to monolayer culture of these cells [14]. Similarly, chondrocytes cultured in a 3D hydrogel with restricted binding sites show prolonged expression of chondrogenic markers compared to standard 2D culture, in which chondrocytes rapidly lose their spherical shape and assume a fibroblastic phenotype [15, 16, 17].

However, many existing 3D models lack precise spatial control over the presentation of instructive (bio)chemical, physical, and dimensional cues to cells [18, 19]. In the case of cells embedded in hydrogels, these approaches have primarily focused on aligning and texturing the cells within the gels. One example is the unidirectional extension of fibroblasts and nerve cells within the anisotropic hydrogel “Anisogel” [20]. This was achieved using magneto‐responsive, cell‐adhesive short fibers that orient in the direction of an external magnetic field. Another example is the alignment of human tendon stem/progenitor cells in a type I collagen hydrogel using iron oxide nanoparticles under a remote magnetic field [21]. Overall, there remains a demand for culture platforms that provide spatial control over cell‐instructive cues, such as the defined distribution of cell‐adhesive ligands.

Microscale patterning techniques have emerged as powerful tools to present biochemical and ‐physical cues to cells in a geometrically controlled manner [22]. By creating micropatterns of ECM proteins or peptides surrounded by low‐protein‐attachment or –binding and cell‐repellent regions on flat surfaces, micropatterning enables precise control over cell adhesion, shape, and behavior [23, 24]. Due to its efficiency, micropatterning has become the most popular method to investigate cell response to specifically defined environmental cues [25]. Landmark studies demonstrated that the geometric control over ECM‐coated adhesive substrates allows to govern cell behavior. For example, the decrease of the size of adhesive ECM islands is responsible for switching human and bovine capillary endothelial cells from growth to apoptosis [26]. Moreover, the spatial distribution of ECM patterns on a substrate plays a determining role in defining the orientation of the division axis of HeLa human cervical epithelial adenocarcinoma cells [27]. Finally, the shape of ECM protein patterns, in addition to their size, affects the differentiation of human mesenchymal stem/stromal cells (hMSCs) toward the adipogenic and osteogenic lineages [28]. High‐throughput platforms, such as the “Galapagos Chip”, have further expanded the capability to explore libraries of adhesive micropatterns and their interactions with cells [29].

**FIGURE 1:**
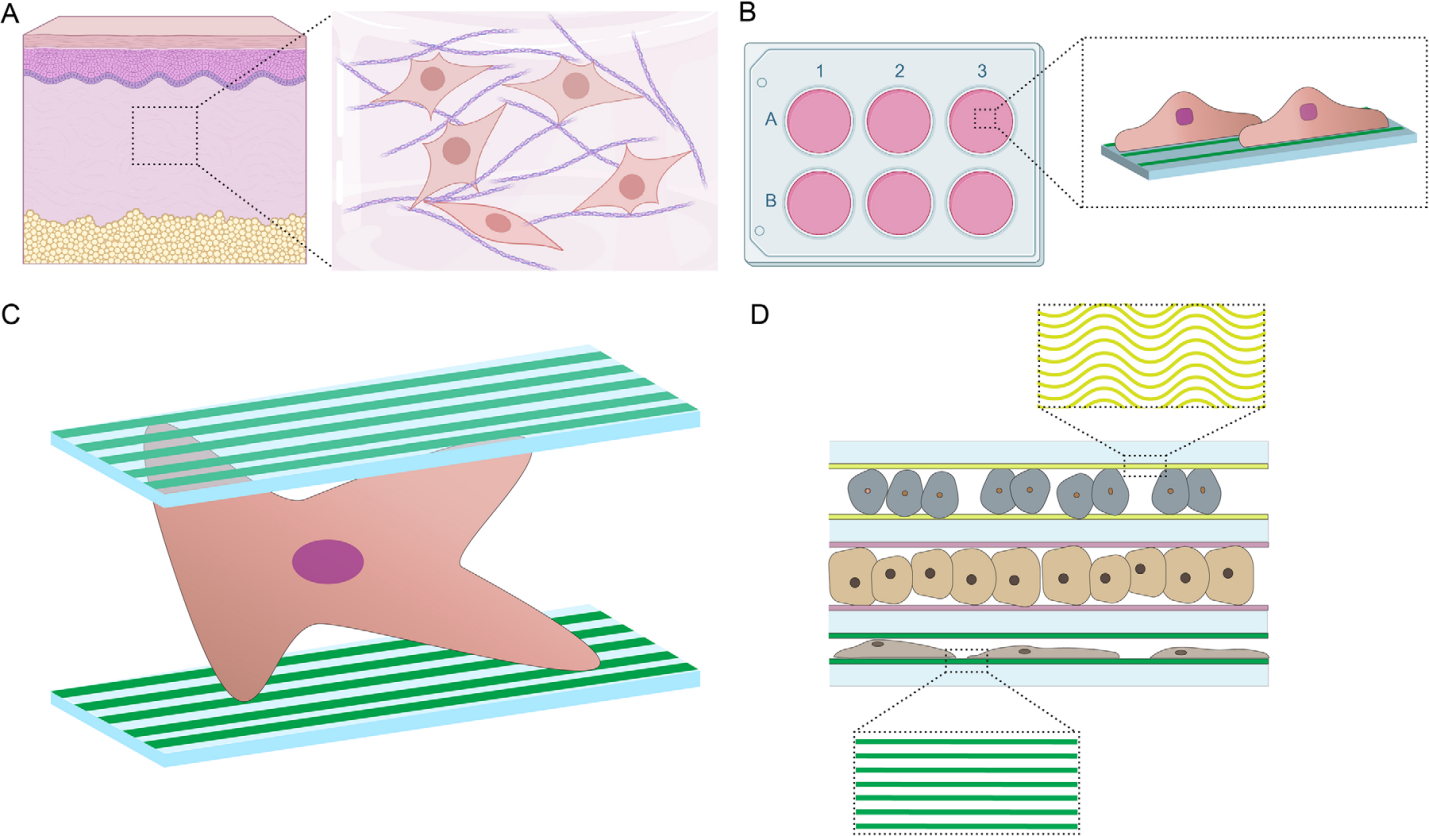
Concept of the FC‐Chip. (A) Cells in tissues often reside in a 3D environment in which they are surrounded by ECM that provides mechanical support, and biochemical and biophysical cues to direct cell fate and function. (B) Cells grown on 2D culture platforms experience non‐physiological conditions because only their ventral surface contacts the culture substrate. This limited interaction fails to replicate the complex 3D environment found in vivo, thereby impacting cellular behavior and compromising the representation of physiological processes. (C) The FC‐Chip utilizes cell‐adhesive cues on two opposing 2D substrates to recreate a pseudo‐3D system that stimulates both the dorsal and ventral sides of cells. This approach mimics more in vivo‐like conditions. (D) The FC‐Chip offers the possibility for multi‐layered tissue‐like constructs. To accommodate the different cell types, each membrane could be patterned with the corresponding ECM proteins. For instance, to create enthesis‐like constructs, tenocytes could be cultured in a first layer, which is enclosed by opposing membrane surfaces micropatterned with wave‐like collagen type I structures. Chondrocytes could be cultured in a second layer enclosed by membrane surfaces uniformly coated with collagen type II. Lastly, osteoblasts could be cultured in a third layer enclosed by membrane surfaces micropatterned with line‐like mineralized collagen type I structures.

While these 2D micropatterning approaches have provided valuable insights into cell‐material and–surface interactions, they fail to capture the 3D nature of many cellular microenvironments. To address this limitation, in this proof‐of‐concept study, we developed the “Floor‐Ceiling‐Chip” (FC‐Chip) (Figure 1). This novel platform includes adhesive cues in the form of micropatterned collagen islands on two opposing, otherwise non‐adhesive 2D substrates, creating a 3D system that stimulates both the dorsal and ventral sides of cells in between. The platform therefore also eliminates artificial cell polarization, for example, of stromal cells, observed in standard culture dishes and inserts. The membranes of the FC‐Chip were provided with micropatterned adhesive islands of varying geometries and sizes, enabling control over cell shape and alignment in a 3D environment. To validate the 3D character of the cellular microenvironment provided by the FC‐Chip, we analyzed nuclear and cellular morphology, focal adhesion size, nuclear translocation of the mechanosensitive transcription factor yes‐associated protein (YAP)1, and lamin A/C expression in single cells.

Taken together, conventional 2D culture systems and substrates, such as the flat bottoms of culture dishes or planar biomaterial sheets, allow complex patterning and (2D) shaping of cells. However, these systems lack the spatial distribution of cell‐adhesive anchoring sites that stromal cells, for example, encounter in vivo. In contrast, 3D culture systems, such as cells embedded in hydrogels, provide access to 3D‐distributed adhesive sites but do not support cell patterning and shaping to the same extent as 2D systems. Patterning cell‐adhesive sites in hydrogels remains challenging in terms of design variability and resolution compared to what is achievable with 2D techniques. To overcome this limitation, we extended the 2D approach by layering it twice. Microfabrication of the 2D building blocks of this 2 × 2D approach, in turn, is routine in most bioengineering and many biology labs. The FC‐Chip takes micropatterning and ‑shaping of cells to a next, “pseudo‐” or “quasi‐” 3D level. With the cells adhering to adhesive patterns on two opposing permeable membranes, the FC‐Chip holds potential for advanced studies of the molecular response of cells to their induced 3D‐like spatial morphology.

## 2 Results and Discussion

### 2.1 Design, Microfabrication, Inoculation, and Assembly of the FC‐Chip

The concept behind the FC‐Chip is based on the integration of two opposing 2D substrates, which enable stimulation of both the dorsal and ventral sides of cells, resulting in a pseudo‐3D platform. In addition, to control spatial cell adhesion and thereby cell shape and alignment, the substrates of the FC‐Chip were provided with micropatterned cell‐adhesive islands (Figure 1).

To ensure compatibility with prolonged cell culture, we first selected a suitable substrate (base) material using a platform‐internal material selection metric under identical culture conditions (substrate coating, cell seeding density, and culture duration). We evaluated the viability of hMSCs cultured in FC‐Chips assembled from uniformly collagen‐coated glass coverslips or porous or non‐porous polycarbonate (PC) membranes and on 2D substrates from uniformly collagen‐coated single glass coverslips or non‐porous PC membranes (Figure S1). The porous membranes were ion track‐etched membranes, as similarly known from culture inserts. During a 10‐day culture period, we measured a significant reduction in cell viability when the FC‐Chips were assembled from coverslips or non‐porous membranes, in contrast to a robust viability observed in conjunction with porous membranes (Figure S1A). In addition, we observed reduced cell adhesion and viability on the 2D glass substrates, potentially due to the instability of the collagen coating on the glass substrates, despite oxygen plasma treatment (Figure S1B). Consequently, we opted for the porous membranes as the substrate material for the FC‐Chip, which ensures the supply of oxygen and nutrients and removal of metabolic waste by diffusion for a sustained long‐term cell culture. Comparable levels of cell viability were achieved with FC‐Chips assembled from membranes with pore sizes of 0.4 and 2 µm. However, the use of 2‐µm pore size membranes more impeded the passage of light during imaging. Hence, membranes with 0.4 µm pore size were selected for the assembly of the FC‐Chips.

The Young's modulus of the membranes was determined from stress‐strain curves measured by tensile testing (Figure S2A,B, respectively) and was found to be 0399 ± 0050 GPa. Due to the porosity of the membranes, this value likely underestimates the actual Young's modulus of the PC material from which the membrane is made (between the pores). At RT and 37°C, PC behaves predominantly elastically, with a high Young's modulus in the low gigapascal range (≳ 2 GPa) [30] and low energy dissipation. In contrast, collagen hydrogels, for example, exhibit pronounced viscoelasticity, with low shear storage moduli typically in the few hundred pascal range [31] and higher loss factors. Even though cell adhesion was observed to be confined to the rather thin collagen micropatterns, the overall perception of substrate stiffness in all directions is likely dominated by the underlying stiff PC membrane. It is important to note, however, that the FC‐Chip approaches the study of mechanotransduction by directly inducing cell shape, rather than relying on the mechanical properties of the culture substrate or embedding gel.

**FIGURE 2:**
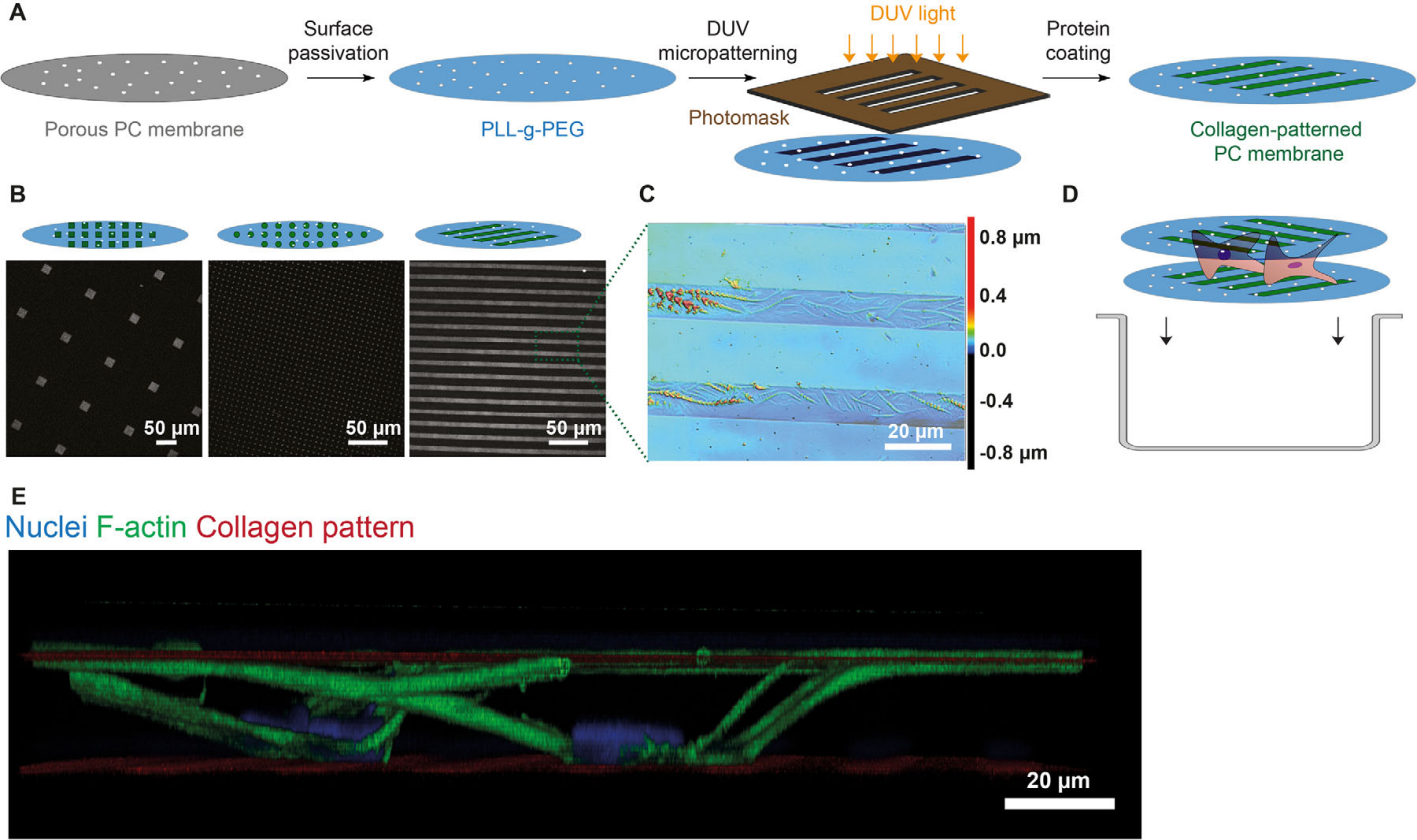
Design and microfabrication of the FC‐Chip. (A) Schematic representation showing the fabrication process, based on porous PC membranes as culture substrates, which were passivated with PLL‐g‐PEG to minimize non‐specific protein binding. A photomask with the desired light absorber patterns was placed over the passivated PC membranes. The membranes were then locally exposed to DUV light, which activated the regions not covered by the mask absorbers. When covering the membranes with a collagen solution, the activated regions were selectively functionalized with this protein. (B) Fluorescence microscopy images of the collagen patterns used in the study, which are squares with 20 µm side length and 100 µm spacing between them (left), dots with 3 µm diameter and 5 µm spacing between them (middle), and lines with 10 µm width and 20 µm spacing between them (right). (C) Optical profilometry image of line‐shaped collagen patterns on a porous PC membrane showing the formation of collagen fibrils within the patterned regions. The color legend indicates the height level. (D) Schematic illustration of the assembly of the FC‐Chip in microwells. (E) Fluorescence confocal microscopy image of hMSCs cultured in the FC‐Chip as depicted in D), showing simultaneous interaction with the dorsal and ventral substrate of the chip. The cells were stained for filamentous (F‐)actin (green) and nuclei (blue).

The opposing surfaces of the substrates were provided with collagen micropatterns using deep UV (DUV) lithography‐based surface modification (Figure 2A) [32]. Briefly, the porous PC membranes were first coated with poly(L‐lysine)‐graft‐poly(ethylene glycol) (PLL‐g‐PEG), a chemistry that inhibits protein adsorption and cell adhesion. The PLL‐g‐PEG‐coated membranes then underwent local DUV irradiation through a photomask, causing degradation and activation of the exposed PLL‐g‐PEG. After covering the membranes with a collagen type I solution, the exposed areas were selectively functionalized with this protein, resulting in homogeneous immobilization of collagen fibrils within these areas (Figure 2B,C). In this study, we employed micropatterns with various feature shapes – including lines, dots, and squares –, dimensions, and spacings between the features to showcase the versatility of the approach (Figure 2B,C; Table S1). Confocal laser scanning microscopy of a porous PC membrane with a collagen micro line pattern showed the formation of collagen fibrils in the patterned regions (Figure S3A). Optical profilometry revealed a roughness of R_z_ = 0.444 µm (Figure S3B).

hMSCs were seeded on the bottom substrate prior to being brought into contact with the top one (Figure 2D). No technical spacer was introduced between the top and the bottom substrate. This seeding and assembly strategy allows cells to establish interactions with the patterned collagen regions present on both the bottom and top substrates (Figure 2E). In doing so, the cells self‐adjust the gap between the two substrates, creating a confined yet permeable space for cell growth, the latter due to the pores in the substrates. A rough rotational alignment of the patterns on the bottom and top substrates (see Section 2.3) was performed manually. A translational alignment of the patterns, whose (horizontal) spacing was in a range similar to that of the (vertical) gap between the bottom and top substrates (Table S1), has not yet been performed in this proof‐of‐concept study.

In comparison to previous studies describing “sandwich culture” platforms [33, 34, 35, 36], the FC‐Chip exhibits advantageous features. First, the use of porous track‐etched membranes facilitates long‐term culture by enabling the diffusion of oxygen and nutrients to the cells in the platform. The cylindrical submicrometer pores, oriented perpendicular to the membrane plane, thereby only minimally interfere with the light passing through during imaging. The porous membranes supported cell viability for at least 10 days. Second, the FC‐Chip incorporates micropatterning of relevant ECM proteins, enabling spatial control of cell adhesion. Unlike previous platforms fabricated with uniformly coated ECM proteins or polystyrene nanogrooves [37], the FC‐Chip offers the potential to directly control cell shape in a pseudo‐3D configuration. The FC‐Chip's features open new possibilities for studying cell behavior in response to specific microenvironments.

### 2.2 Micropatterned Cell‐Adhesive Islands Enable Control of 3D Cell Morphology in the FC‐Chip

The geometries and sizes of cell‐adhesive micropatterns can have different effects on cell functions [23, 38]. Adhesive micropatterns at the cell‐scale on otherwise non‐adhesive substrates provide a means to spatially confine cells and thus control their shape within a specific geometry [39]. By manipulating cell shape and size through micropatterning, it became possible to influence cell survival of human and bovine capillary endothelial cells [26] and direct the differentiation of hMSCs [28].

The translation of these findings to the third dimension has hardly been investigated yet. Culturing cells in open [40, 41] and lid‐covered closed [42, 43] single‐cell microwells are other approaches that enable the control of cell shape in 3D. However, the open wells cause the cells to have a non‐uniform shape toward the well opening, and the cells polarize due to a lack of integrin‐binding from the dorsal side. In microwells closed by a (cell‐adhesive) lid, there is more control of the shape of the cells [42, 43]. Similar to the closed microwells, we hypothesized that the FC‐Chip could control cell shape in 3D and enable cell adhesion on the dorsal and ventral sides.

**FIGURE 3:**
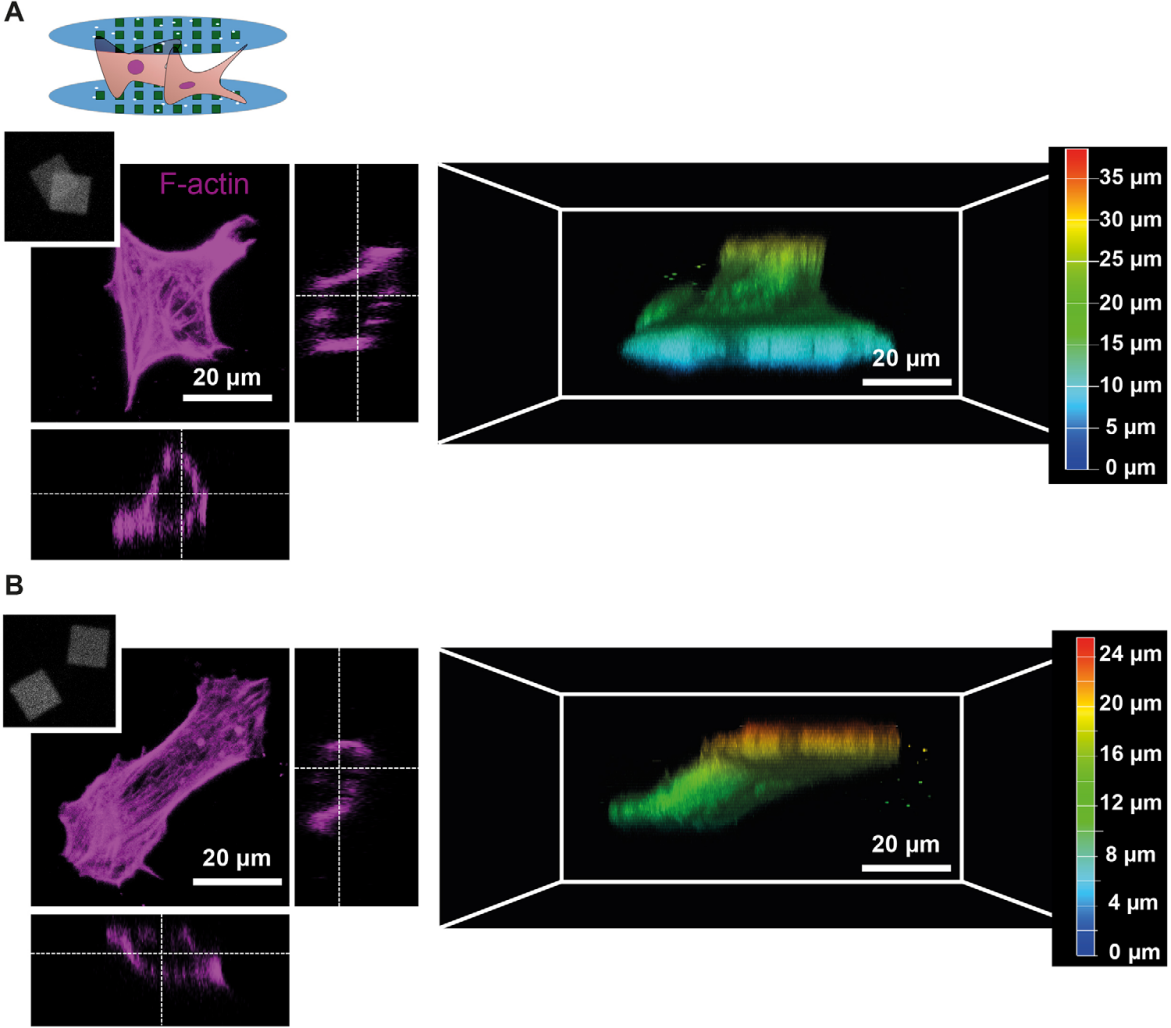
Manipulation of cell morphology. (A) Fluorescence confocal microscopy images (group of three similar images on the left) and corresponding 3D reconstruction (right) of hMSCs cultured for 24 h in the FC‐Chip assembled from membranes carrying aligned 20 µm square collagen patterns with overlap between the patterns of the two substrates, see upper left inset; the inset shows a maximum intensity projection of label‐free fluorescence microscopy images of in each case one collagen pattern on the two stacked substrates, obtained using excitation at 488 nm. The cells were stained for F‐actin (magenta). The scale bar on the left applies to all images of the subfigure except the inset. The color legend indicates the height level. (B) Fluorescence confocal microscopy images (group of three similar images on the left) and corresponding 3D reconstruction (right) of hMSCs cultured for 24 h in the FC‐Chip assembled from membranes carrying non‐aligned 20 µm square collagen patterns with space between the patterns of the two substrates, see upper left inset; the inset shows a label‐free fluorescence microscopy image of in each case one collagen pattern on the two stacked substrates, obtained using excitation at 488 nm. The cells were stained for F‐actin (magenta). The scale bar on the left applies to all images of the subfigure except the inset. The color legend indicates the height level.

To enable the manipulation of cell adhesion to the opposing surfaces of the two substrates of the FC‐Chip, we designed square‐shaped collagen micropatterns with a side length of 20 µm and a spacing of 100 µm between them, preventing cells to bridge adjacent patterns on the bottom or top substrate (Figure 3). We then assembled the micropatterned membranes and identified two different configurations, aligned and not aligned, that is, with or without overlap of the patterns of the bottom and top substrate. After a 24 h culturing period, hMSCs adhered to the collagen patterns on both the top and bottom substrates of the FC‐Chip, resulting in distinct cell morphologies, apart from the shape of the adhesive islands of the top and bottom substrates, determined by their positioning relative to each other. When the collagen micropatterns were aligned or overlapping, the cells assumed a semi‐pyramidal shape, suggesting an adaptation to the geometry of the micropatterns (Figure 3A). In contrast, when spaced micropatterns were present, the cells exhibited a more elongated shape and formed bridges between the top and bottom substrates of the FC‐Chip (Figure 3B). This behavior indicates the ability of cells to adapt and respond to the spatial arrangement of the micropatterns, resulting in different 3D cell morphologies.

Smaller, subcellular‐scale micropatterns in the form of three different dot‐shaped collagen micropatterns with a fixed diameter of 3 µm and varied spacings of 5, 10, or 20 µm revealed a certain limitation in manipulating cell shape in 3D. In the first trials with the current, not fully optimized setting up of the culture in the FC‐Chip, also compared to the bottom substrate, the cell interaction with the top substrate seemed rather limited, even if the cells were still found adapting 3D morphologies (Figure S4). This was likely due to the small size of the adhesive islands and also to the sparse nature of the patterns in terms of the ratio of adhesive area to total area. This is also why this study was primarily conducted with the larger and less sparse, denser line patterns.

### 2.3 Cytoskeletal Remodeling and Cell‐Morphological Changes in the 3D Microenvironment of the FC‐Chip

**FIGURE 4:**
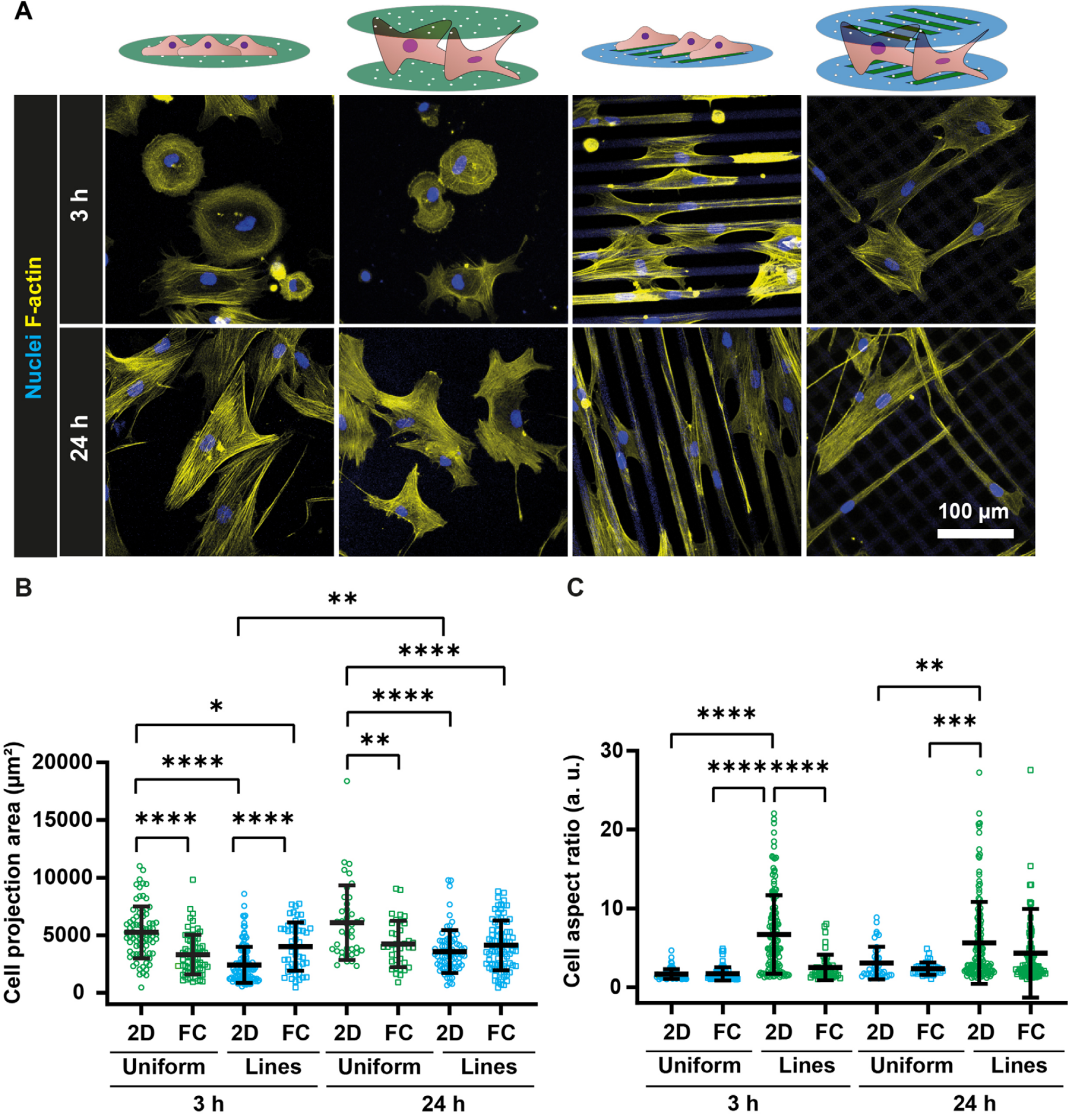
Cell morphology. (A) (Maximum intensity projection of) fluorescence confocal microscopy images of hMSCs cultured for 3 (top row) and 24 h (bottom row) on uniformly collagen‐coated 2D substrates (first column) or FC‐Chips assembled from the same substrates (second column) and on collagen line‐patterned 2D substrates (third column) or FC‐Chips assembled from the same substrates (fourth column). The cells were stained for F‐actin (yellow) and nuclei (blue). The scale bar applies to all images of the subfigure. (B) Quantification of cell area in cells cultured for 3 and 24 h on the different 2D substrates and corresponding FC‐Chips. The long horizontal lines and error bars represent the median values and interquartile ranges, respectively. Significance was determined by one‐way ANOVA followed by Tukey's post‐hoc test ^*^ *p* < 0.05, ^**^ *p* < 0.01, and ^****^ *p* < 0.0001. N = 3. (C) Quantification of cell area in cells cultured for 3 and 24 h on the different 2D substrates and corresponding FC‐Chips. The long horizontal lines and error bars represent the median values and interquartile ranges, respectively. Significance was determined by one‐way ANOVA followed by Tukey's post‐hoc test ^**^ *p* < 0.01, ^***^ *p* < 0.001, and ^****^ *p* < 0.0001. N = 3.

The cellular cytoskeleton contains a network of protein filaments responsible for maintaining cell shape and providing mechanical support [44]. Changes in cell area reflect alterations in cytoskeletal organization and cell‐substrate interactions [45, 46], and if manipulated by patterning cell‐adhesiveness can provide valuable information about the response of cells to their microenvironment. To evaluate the cytoskeletal adaptation to the FC‐Chip microenvironment, using fluorescence microscopy images, we monitored the (cell) area and aspect ratio of hMSCs after 3 and 24 h of culturing in FC‐Chips from uniformly collagen‐coated or line‐patterned membranes and on corresponding 2D controls (Figure 4). Cells cultured on line‐patterned substrates showed alignment along the collagen adhesive regions, in both 2D and FC‐Chips and at both time points (Figure 4A).

After 3 h of culturing, the cells showed a significantly higher cell area when cultured on uniformly coated 2D substrates compared to the other conditions analyzed at the same time point, a relation that remained the same at the 24 h time point (Figure 4A,B). We measured a significantly higher cell area at 3 h in FC‐Chips with line patterns, (manually) perpendicularly aligned to each other, relative to their 2D controls. The observed increase in cell area might be attributable to the necessary reorientation of the cells between the opposing substrates. A similar cell behavior was reported in another study on a sandwich culture system [37].

Cell aspect ratio, which is a measure of cell elongation, was significantly higher in cells cultured on line‐patterned 2D substrates compared to the other conditions after 3 h of culturing (Figure 4A,C). This is in line with data on cell area discussed in the last paragraph, demonstrating the confinement of cells on the protein‐coated adhesive regions. The significantly reduced aspect ratio in FC‐Chip cultures relative to cultures on 2D controls with line patterns might be a further indication of cell spreading in the third dimension, inducing cells to also attach to the collagen patterns on an opposing substrate. After 24 h, the cells showed significantly higher elongation on membranes with the 2D line pattern compared to uniformly coated membranes in both 2D and the FC‐Chip, while comparable values were measured with FC‐Chips with the line pattern. These results are similar to a previous study that reported a decrease in cell alignment and elongation in sandwich culture conditions compared to 2D substrates in combination with nanogroove patterns [37, 47].

**FIGURE 5:**
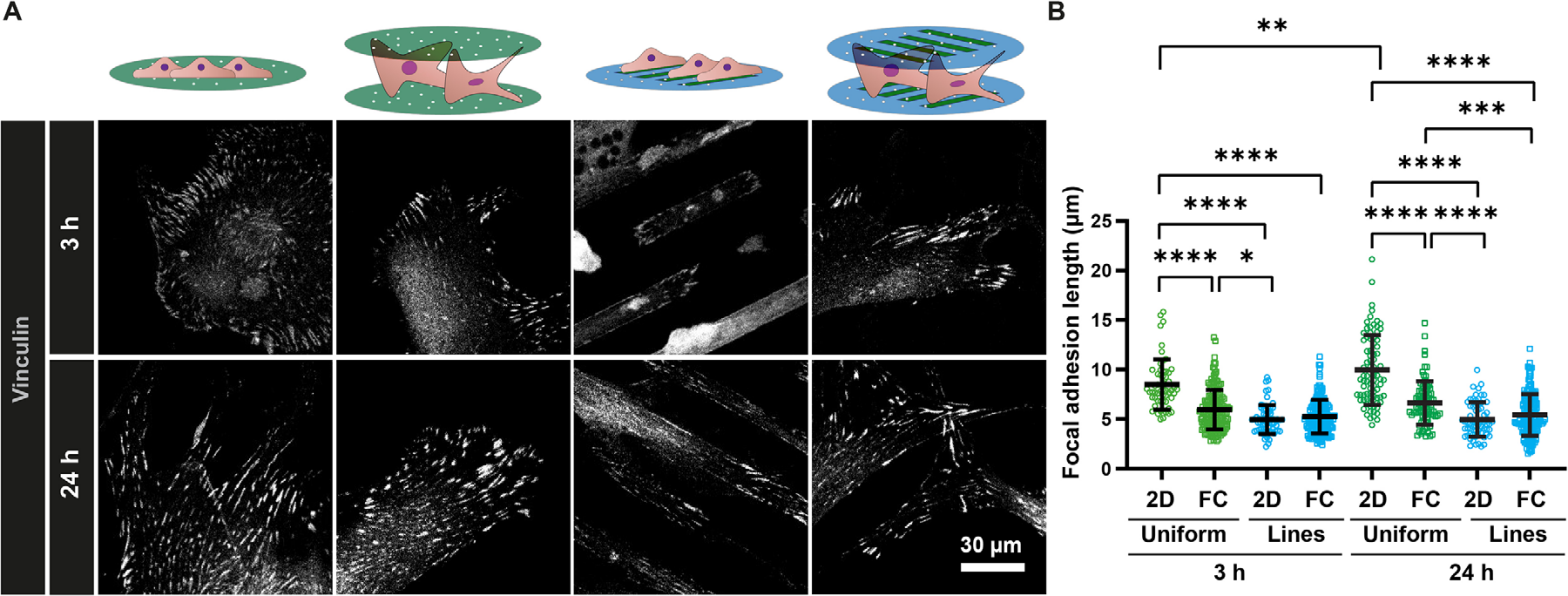
Focal adhesion length. (A) Fluorescence confocal microscopy images of hMSCs cultured for 3 (top row) and 24 h (bottom row) on uniformly collagen‐coated 2D substrates (first column) or FC‐Chips assembled from the same substrates (second column) and on collagen line‐patterned 2D substrates (third column) or FC‐Chips assembled from the same substrates (fourth column). The cells were stained for vinculin (grey). The scale bar applies to all images of the subfigure. (B) Quantification of focal adhesion length in hMSCs cultured for the different time points on the different substrates. The long horizontal lines and error bars represent the median values and interquartile ranges, respectively. Significance was determined by one‐way ANOVA followed by Tukey's post‐hoc test. ^*^ *p* < 0.05, ^**^ *p* < 0.01, ^***^ *p* < 0.001, and ^****^ *p* < 0.0001. N = 3.

Based on the observed changes in cell morphology, we investigated whether the FC‐Chip influenced the formation of focal adhesions (Figure 5), a critical component of mechanotransduction [2]. Therefore, from fluorescence microscopy images (Figure 5A), we quantified the size of vinculin‐containing focal adhesions in hMSCs cultured in FC‐Chips assembled from uniformly collagen‐coated or line‐patterned substrates as well as on corresponding 2D controls, both after 3 and 24 h of culture (Figure 5B). At the earlier time point, we observed a significant increase in the focal adhesion length in cells cultured on uniformly collagen‐coated 2D substrates compared to the other conditions analyzed. This trend persisted at the later time point. Then, we also observed a significant decrease in focal adhesion length in the FC‐Chip assembled from line‐patterned substrates compared to the FC‐Chip from uniformly coated substrates. However, cultures on FC‐Chip from line‐patterned substrates exhibited shorter focal adhesions than cultures on 2D substrates, which indicates less maturation [48], similar to observations made in cultures on confined micropatterns. Additionally, the presence of smaller focal adhesions suggests lower cellular tension, resembling characteristics of cells cultured within 3D matrices [49]. We additionally embedded hMSCs in a 3D collagen gel (Figure S5). The vinculin staining in the collagen gel was not quantified due to limitations in reliably segmenting adhesion structures in volumetric images. However, qualitative assessment revealed that, similar to the FC‐Chip from uniformly coated substrates, vinculin‐rich adhesion structures were less prominent in the collagen gel compared to the uniformly collagen‐coated 2D substrate. These findings indicate that cells cultured in the FC‐Chip can respond to the spatial organization of its adhesive patterns by maturation and adjustment of the length of focal adhesions, reflecting their ability to perceive and adapt to the 3D microenvironment. The decreased focal adhesion length observed in FC‐Chip cultures also aligns with previous observations in sandwich culture platforms [33].

### 2.4 Exploring Mechanobiology in the FC‐Chip

**FIGURE 6:**
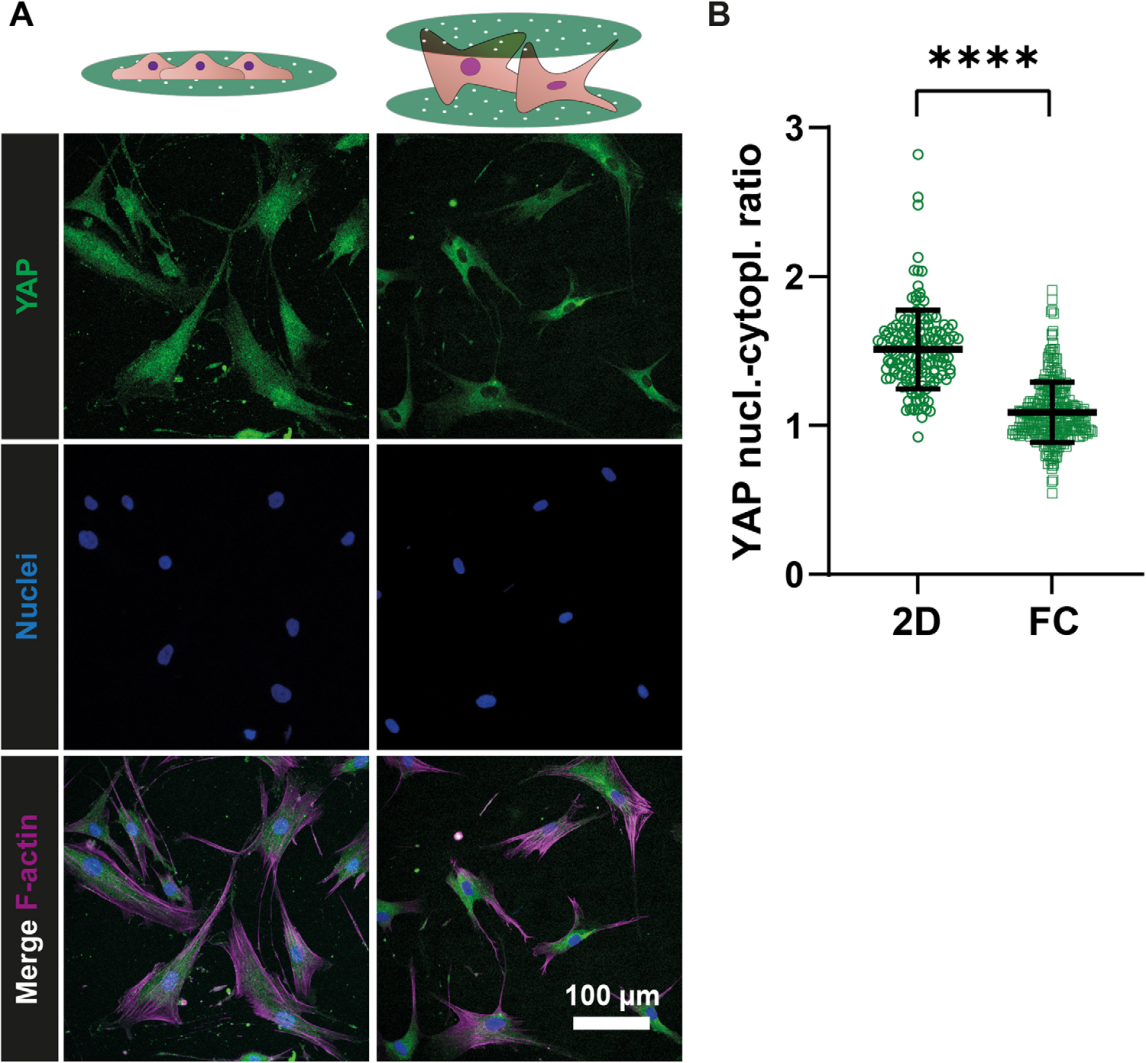
YAP1 cellular expression. (A) Fluorescence confocal microscopy images of hMSCs cultured for 24 h on uniformly collagen‐coated substrates in 2D and corresponding FC‐Chips and stained for YAP1 (green), F‐actin (purple), and nuclei (blue). The scale bar applies to all images of the subfigure. (B) Quantification of YAP1 nuclear‐cytoplasmic ratios in 2D and FC‐Chip culture conditions. The long horizontal lines and error bars represent the median values and interquartile ranges, respectively. Significance was determined by Student's t‐test. ^****^ *p* < 0.0001. N = 3.

YAP(1) is a transcriptional regulator that responds to mechanical cues and is sensitive to various mechanical stimuli [50], including ECM rigidity, strain, shear stress, and cell‐adhesive area [51]. It has been previously demonstrated that YAP expression in hMSCs is affected by dimensionality, showing a significantly lower nuclear‐cytoplasmic ratio of YAP in 3D conditions compared to 2D [52, 53]. Therefore, to investigate the dimensionality perceived by cells cultured in the FC‐Chip, from fluorescence microscopy images (Figure 6A), we measured the nuclear translocation of YAP1 in hMSCs cultured for 24 h on FC‐Chips assembled from uniformly collagen‐coated membranes and corresponding 2D controls (Figure 6B). The YAP1 nuclear‐to‐cytoplasmic ratio was significantly lower in the FC‐Chip cultures relative to the 2D controls. A similarly significant reduction was also observed in 3D collagen gel cultures (Figure S6). These results indicate that more YAP1 remained in the cytoplasm than was translocated to the nucleus. This may be due to the potentially lower tension experienced by the cells (see last section) and the nucleus in the FC‐Chip, which could contribute to preventing YAP1 from entering the nucleus and increasing its cytoplasmic retention [53, 54, 55]. In the FC‐Chip, the lower nuclear translocation of YAP1 in hMSCs can also be attributed to the regulation of the Hippo pathway by globular (G‐)actin levels [56]. The reduction of cellular tension in 3D conditions corresponds to decreased actin polymerization, leading to increased G‐actin levels. The increase in G‐actin promotes Hippo pathway activation, retaining YAP1 in the cytoplasm [57].

**FIGURE 7:**
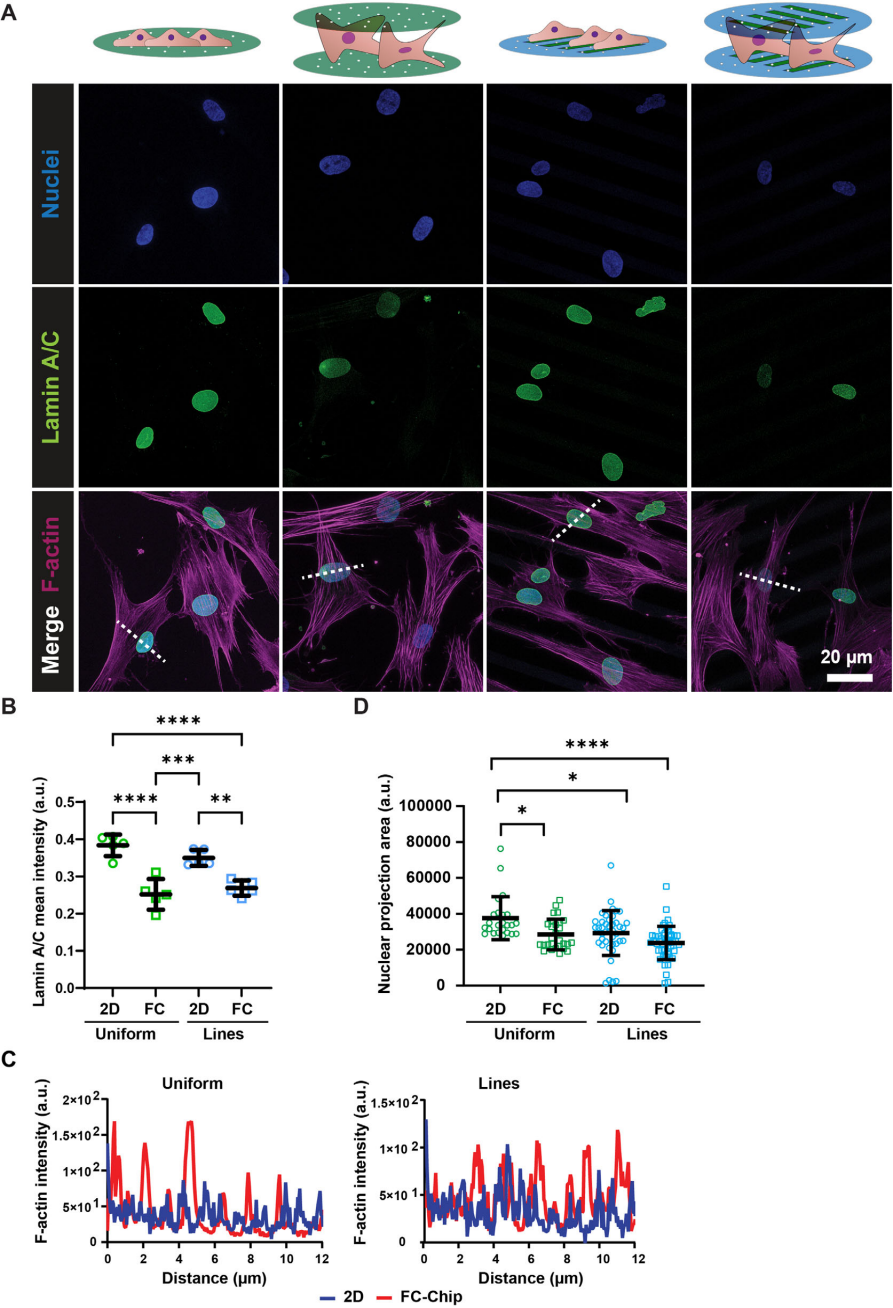
Lamin A/C expression. (A) Fluorescence confocal microscopy images of hMSCs cultured for 24 h on uniformly coated and line–patterned membranes in 2D and FC‐Chip culture conditions. The cells were stained for nuclei (blue), lamin A/C (green), and F‐actin (purple). The scale bars represent 20 µm. (B) Quantification of the mean fluorescent intensity of lamin A/C expressed in cells cultured on uniformly collagen‐coated 2D substrates (first column) or FC‐Chips assembled from the same substrates (second column) and on collagen line‐patterned 2D substrates (third column) or FC‐Chips assembled from the same substrates (fourth column). a.u. … arbitrary units. The long horizontal lines and error bars represent the median values and interquartile ranges, respectively. Significance was determined by one‐way ANOVA followed by Tukey's post‐hoc test. ^*^ *p* < 0.05 and ^****^ *p* < 0.0001. N = 5. (C) Quantification of the distribution of the nuclear F‐actin fluorescence intensity across the nucleus in cells cultured on uniform collagen‐coated (left) and line‐patterned membranes (right). The peaks in the intensity graphs indicate actin stress fibers. N = 3. Each red and blue graph is only from one representative nucleus, and the graphs of the other two quantified nuclei in each case are not shown. a.u. … arbitrary units. (D) Quantification of the nuclear area. The long horizontal lines and error bars represent the median values and interquartile ranges, respectively. Significance was determined by one‐way ANOVA followed by Tukey's post‐hoc test. ^*^ *p* < 0.05 and ^****^ *p* < 0.0001. N = 3. Per independent sample, 10 cells were analyzed.

To further prove that cells cultured in the FC‐Chips exhibit similar responses to those observed in a 3D environment, from fluorescence microscopy images (Figure 7A), we evaluated the expression of lamin A/C, a protein that assists in linking actin filaments to the nucleus [58], in chips assembled from uniformly collagen‐coated or collagen line‐patterned substrates and corresponding 2D controls (Figure 7B). The 2D controls revealed a significantly higher mean fluorescent intensity of lamin A/C expressed in cells compared to the FC‐Chip cultures. In addition, lamin A/C levels in uniformly collagen‐coated FC‐Chips were closer to those observed in the 3D collagen gels than in the 2D controls (Figure S7), supporting a more 3D‐like nuclear mechanoadaptation in the FC‐Chip condition. This result is in line with a previous study demonstrating that in 3D scaffolds hMSCs showed lower lamin A/C expression compared to 2D substrates [53]. Similarly, lamin A levels are increased in cells cultured on stiff 2D materials [59], and while on convex surfaces lamin A/C levels increase, they decrease on concave surfaces, depending on the force distribution within the cell [60].

Lamins are involved in the transmission of mechanical signals from the extracellular microenvironment to the cell nucleus [61], and are mechanically coupled to actin stress fibers that protect the chromatin content of the nucleus against forces on the nuclear membrane [62]. Therefore, we quantified the distribution of the F‐actin filaments across the cell nucleus (Figure 7A,C). When hMSCs were cultured in FC‐Chips, we observed fewer stress fibers overlapping the nucleus compared to 2D controls. The absence of such many stress fibers is a sign of lower cellular tension, as previously shown for hMSCs cultured in 3D [53]. The link between lamin A/C and the actin cytoskeleton was previously described in 2D systems [63, 64]. However, the signaling pathway involved in determining the molecular interplay in 3D environments is still unknown.

Since lamins have a determinant role in maintaining the nuclear shape, we then quantified the size of the nucleus (Figure 7A,D). Cell nuclei in FC‐Chips assembled from uniformly collagen‐coated substrates showed a significant 25% decrease in nuclear area relative to their 2D controls. For line‐patterned substrates, there was a similar trend. This result is in line with previous studies, showing a decrease in nuclear cross‐sectional area between cells under 2D and 3D conditions [55, 65]. Mechanistically, this discrepancy might be related to the higher presence of actin stress fibers around the nucleus in 2D cultures, which generate contractile forces that flatten the nucleus and increase its cross‐sectional area [66]. In contrast, reduced cytoskeletal tension in the 3D environment allows the nucleus to adapt a more rounded shape with a smaller cross‐sectional area. The contractile forces in 2D also induce nuclear pore stretching, promoting YAP nuclear translocation. Surprisingly, the analysis of the nuclear area on collagen line‐patterned substrates showed no significant difference between 2D and FC‐Chip cultures (Figure 7C). This observation might be related to the reduced cell‐substrate contact area imposed by the collagen micropatterns. The width of the cell‐adhesive micropatterns, the 10 µm wide lines, is close to the subcellular range, inducing cells to adapt their shape to the micropattern scale. In accordance with this, we observed a decrease in cell area on line‐patterned substrates compared to uniformly collagen‐coated ones.

Overall, in the 2D culture environment, hMSCs exhibit an increased cell area, facilitating the formation of large focal adhesions (Figure S8). Similarly, we have observed that tenocytes grown on topographically patterned surfaces, compared to flat surfaces, display less F‐actin, as well as smaller and fewer focal adhesions [67]. This phenomenon is accompanied by an upregulation of lamin A/C expression, resulting in larger nuclei areas and increased retention of YAP1 within the cell nuclei. The presence of large focal adhesions and high lamin A/C expression allows for the formation of actin stress fibers that overlap the cell nuclei. On the other hand, in the FC‐Chip, cellular tensions are distributed in multiple directions, leading to small and few focal adhesions. The reduction in focal adhesion size results in a downregulation of lamin A/C expression. Consequently, the nucleus size becomes smaller, and the retention of YAP1 decreases. In the FC‐Chip, the combination of small and few focal adhesions along with low lamin A/C expression inhibits the formation of stress fibers that overlap the nuclei.

### 2.5 Osteogenic Differentiation in the FC‐Chip

**FIGURE 8:**
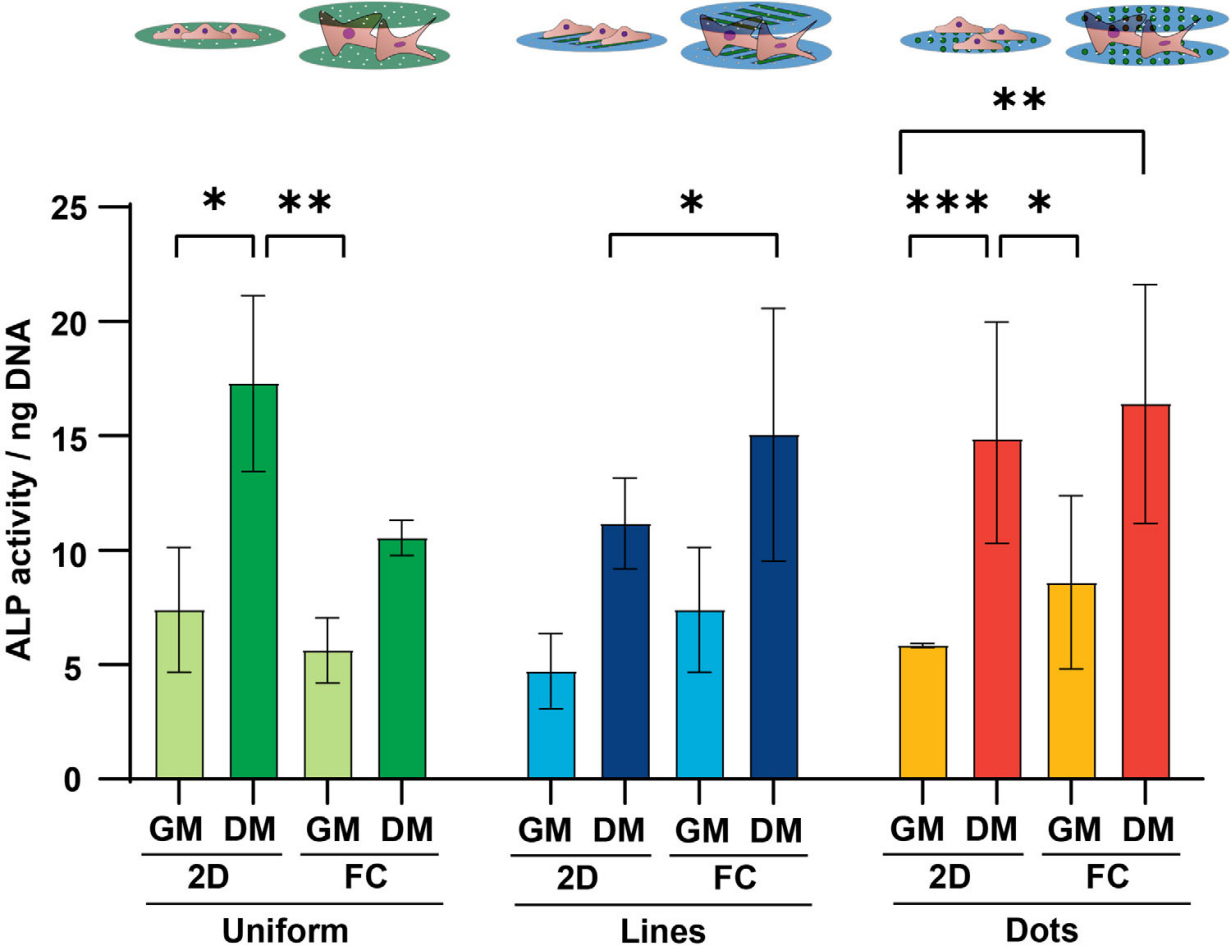
ALP activity. Quantification of ALP activity of hMSCs cultured for 10 days on uniformly collagen‐coated and collagen line‐ and dot‐patterned 2D controls, the latter with 10 µm spacing, and in corresponding FC‐Chips in GM and DM. ALP values were normalized to the DNA content. Bars represent mean values and error bars standard deviations. Significance was determined by one‐way ANOVA followed by Tukey's post‐hoc test. ^*^ *p* < 0.05, ^**^ *p* < 0.01, and ^***^ *p* < 0.001. N = 3. GM … growth medium; DM … differentiation medium.

Since previous studies demonstrated that 3D environments improved osteogenic differentiation [68, 69], we assessed this differentiation in the FC‐Chip environment. For this, we quantified the alkaline phosphatase (ALP) activity in cells cultured for 10 days in FC‐Chips assembled from uniformly collagen‐coated, and line‐ and dot‐patterned substrates and on corresponding 2D controls in either growth medium (GM) or osteogenic differentiation medium (DM) (Figure 8). The data were normalized to the DNA content (Figure S9). As expected, we measured higher ALP activity in samples exposed to DM compared to GM, with a significant increase on uniformly coated and dot‐patterned substrates in 2D conditions. However, in FC‐Chips with the line pattern, we measured an increase in ALP activity in samples exposed to DM approaching a statistically significant difference compared to corresponding 2D controls.

Most importantly, this experiment showed the compatibility of the FC‐Chip with long‐term cultures. Spatial confinement in 3D in vitro and in vivo conditions restricts the diffusion of oxygen and nutrients. This could not only induce a hypoxic environment, affecting cellular metabolism and function, but also limit nutrient delivery, resulting in cell starvation. Furthermore, the confinement of cells in 3D environments can lead to the accumulation of cytotoxic metabolic waste products generated by the cells, compromising cell viability. Our results show that cells cultured in the FC‐Chip receive enough nutrients to proliferate, migrate, and differentiate, ruling out drastic hypoxic, starvation, or toxic effects. The use of porous membranes in combination with the formation of a cell‐induced and self‐adjusted gap between them enables the establishment of a more physiologically relevant culture environment. It ensures a continuous supply of nutrients, oxygen, and signaling molecules to the cells, and the removal of waste products from the cells and gap.

## 3 Conclusion and Outlook

In conclusion, in this proof‐of‐concept study, we present the FC‐Chip as an innovative culture platform to address artificial polarization commonly observed in standard 2D cell culture systems. In addition, the FC‐Chip provides spatial control over environmental cues presented to cells, which is lacking in traditional 3D cultures. The FC‐Chip incorporates two opposing 2D substrates, creating a 3D platform that enables the stimulation of both the dorsal and ventral sides of cells residing between the substrates. By utilizing micropatterning techniques to create cell‐adhesive regions consisting of relevant ECM proteins, we demonstrate the possibility to manipulate cell morphology and behavior. Additionally, despite the physical confinement imposed by the FC‐Chip, it offers compatibility with long‐term cultures. The porous design of the FC‐Chip substrates ensures that medium components and soluble factors remain accessible to the cells throughout the culture period. This compatibility with long‐term cultures is crucial for studying processes over extended periods, such as cell differentiation or tissue development.

An advantage of the FC‐Chip platform is its simple and straightforward (micro)fabrication, which relies on readily available laboratory materials and does not require complex equipment or specialized expertise. Its application in cell culture is similarly easy. This accessibility and simplicity make the FC‐Chip an attractive option for researchers seeking to adopt 3D culture models without facing the challenges often associated with more complicated systems.

Looking out on future developments of the platform, these could include incorporating patterns of distinct proteins on the dorsal and ventral substrates of the FC‐Chip or stacking more than two membranes and cells in between, to model, for example, (graded) connective tissue interfaces. Another logical development would be the extension of the palette of applicable patterning techniques toward microcontact printing. Finally, a meaningful but technically also more demanding further development of the platform would be the precise translational and rotational alignment of the patterns on the bottom and top substrates. This could be achieved by, for example, making use of mechanical stops of square cut substrates both during patterning and culture in x‐ and y‐direction. Bottom and top substrates that are precisely aligned or intentionally misaligned by defined offsets in a controlled manner would then allow to reduce and assess morphology variations across a larger number of cells and enable a more systematic study of how the spatial distribution of cell‐adhesive sites across the two substrates influences cellular behavior. It would also allow upright and vertical or inclined columnar shaping of cells between and by opposing adhesive islands of identical or different geometry and size. The islands could thereby be rotated or shifted against each other. A preview of such pseudo‐3D morphologies can already be seen in Figure 3 of the manuscript.

## 4 Experimental Section

### 4.1 Assembly of the FC‐Chip

The FC‐Chip was fabricated using 50 µm thin dense and ion track‐etched porous PC films or membranes (it4ip, Belgium) as culture substrates. The porous ones had a pore size of 0.4 µm, unless otherwise stated, and a pore density of 10^6^ cm^−2^. Together with coverslips (Epredia, Fisher Scientific; 10 mm diameter), the substrates were assembled in 48‐well plates or, for improved imaging, 35 mm glass bottom dishes (Nunc, Thermo Scientific; catalog number: 150680; with 12 mm diameter glass bottom). Depending on whether the PC membranes were used in the well plate or the dish, they were laser‐cut into 10‐ and 11‐mm diameter circles, respectively (Agora, the Netherlands). Alternatively, sharp (biopsy) punches could be used.

### 4.2 Tensile Testing of porous PC membranes

The mechanical properties of the PC membrane were assessed using a tensile testing machine (ElectroForce 3200‐Series III multiaxial mechanical tester, TA Instruments). The PC membrane was punched into a dog‐bone shape using a stainless‐steel punch and mounted in the mechanical tester using custom‐made titanium grips. Uniaxial tensile tests were performed according to ISO 527‐1 guidelines at a strain rate of 0.01 s^−1^, while the accumulated load was recorded using a 45 N (∼ 4.5 kg weight equivalent) load cell at 50 points s^−1^ until sample failure. Stress‐strain curves were generated by converting the measured force and displacement values into their respective stress and strain values. The curves were smoothed using a moving average calculation (50 points per value representing 1 s of measurement). The tensile Young's moduli were calculated from the linear elastic region of each curve.

### 4.3 Micropatterning of PC Membranes

Micropatterning on the PC membranes was performed adapting the protocol from Azioune et al. [32]. Briefly, the cut membranes were first cleaned with ethanol and distilled water in an ultrasonic bath for 10 min. Then, the membranes were air‐dried and treated with oxygen plasma (PlasmaFlecto 10, plasma technology, Germany) for 3 min at 60 W and 30 mPa. Subsequently, the treated membranes were incubated with 0.1 mg ml^−1^ PLL‐g‐PEG (SuSoS, Switzerland) in 10 mM N‐2‐hydroxyethylpiperazine‐N‐2‐ethane sulfonic acid (HEPES; Thermo Fischer Scientific) for 1 h at room temperature (RT). The PEGylated substrates were rinsed with distilled water and mounted under a quartz photomask (Delta Mask, Netherlands) when still wet for close contact between mask and substrates. The mask‐covered substrates were exposed to DUV light (UV Ozone Cleaner, PSD‐UV, Novascan Technologies, USA) for 15 min. The resulting patterned substrates were kept in 70% ethanol until further use. Prior to cell seeding, the patterned membranes were sterilized with 70% ethanol for 30 min and washed twice with sterile phosphate‐buffered saline (PBS). To enable local cell adhesion, the patterned membranes were selectively coated with collagen (PureCol, Advanced BioMatrix; bovine type I collagen; 3 mg ml^−1^; pH 2) using a 5 µg ml^−1^ collagen solution in PBS. 300 µl of collagen solution was added to the patterned membranes and incubated in sterile conditions for 1 h at RT. Afterwards, the remaining liquid was aspirated, and the coated surfaces were carefully rinsed with PBS and used for cell culture.

For non‐patterned, uniform collagen‐coated membranes and coverslips, they were first treated with oxygen plasma for 3 min at 60 W and 30 mPa, and then sterilized with 70% ethanol for 30 min. The substrates were washed twice with PBS prior to applying the collagen coating, as similarly described in the last paragraph.

### 4.4 Surface Profile Analysis

The surface roughness of the micropatterned films was assessed using a 3D confocal laser scanning microscopy‐based optical profilometer (VK‐X250, KEYENCE, Japan) in combination with the MultiFileAnalyzer analysis software (KEYENCE). For this, the value of Rz, measured and calculated as the (averaged) difference between the tallest peak and the deepest valley in the surface, was used.

### 4.5 Cell Culture

hMSCs were isolated as previously described from bone marrow aspirate of a (single) donor [70] who had given consent, based on a corresponding document from the Medical Ethical Committee (Medisch Ethische Toetsingscommissie; METC) of the Medisch Spectrum Twente hospital, Enschede, The Netherlands (study protocol “Functioneel weefselherstel met behulp vanuit beenmerg verkregen stamcellen”; K06‐002). The cells were expanded in GM, consisting of Minimum Essential Medium (MEM)‐α without nucleosides and of GlutaMAX (Gibco, Thermo Fisher Scientific), and supplemented with 10% (v/v) fetal bovine serum (FBS; Sigma–Aldrich, batch number: BCBT6987), 0.2 mM ascorbic acid (Sigma–Aldrich), 100 U ml^−1^ penicillin‐streptomycin (Thermo Fisher Scientific), at 37°C in a humidified atmosphere with 5% CO_2_. DM was prepared by supplementing GM with 100 nM dexamethasone (Sigma–Aldrich) and 10 mM β‐glycerophosphate (Sigma–Aldrich).

After reaching a confluence of 70%–80%, the cells at passage 3 or 4 were washed with PBS and detached using 0.05% trypsin‐ethylenediaminetetraacetic acid (EDTA) (Thermo Fisher Scientific). Then, the trypsin was neutralized with the addition of GM, the cell suspension was centrifuged at 300 rcf for 5 min, and the cells were resuspended in GM. The cells were seeded on the ventral patterned or non‐patterned PC membranes or glass coverslips at a density of 5000 cells cm^−2^. The samples were then placed in an incubator at 37°C with 5% CO_2_ for 30 min to allow adhesion of the cells to the substrates. Subsequently, for the FC‐Chips, the dorsal substrates were placed on top of the cells, and 10 × 10 × 1 mm^3^ glass substrates on top of the dorsal substrates. This was followed by the addition of 500 µl of culture medium. After allowing the cells to settle and adhere for 30 min, the glass substrates were removed from the top of the FC‐Chips. For 3D controls, hMSCs were embedded in a collagen type I hydrogel. A collagen solution (final concentration: 5 mg mL^−1^; FibriCol, Advanced BioMatrix) was prepared by mixing collagen with 10× PBS and adjusting the pH to 7.0 using sterile 1 M NaOH, followed by dilution with sterile water to the final concentration. The cells were resuspended in the collagen solution at a final density of 5000 cells mL^−1^, and 100 µL of the cell‐laden gel was dispensed per well into a 96‐well glass‐bottom plate. The gels were incubated at 37°C for approximately 40 min to allow polymerization, after which 100 µL of GM was added on top of each gel.

### 4.6 Live/Dead Assay

Cell viability was analyzed using the LIVE/DEAD Viability/Cytotoxicity Kit (Thermo Fisher Scientific) according to the manufacturer's protocol. Briefly, hMSCs were cultured in 2D and FC‐Chip conditions for 10 days, washed twice with PBS, and then incubated with 2 µM Calcein‐AM and 6 µM ethidium homodimer (EthD)‐1 in PBS for 30 min at 37°C in the dark. After washing three times with PBS, the cells were analyzed by fluorescence microscopy (Eclipse TS100 microscope, Nikon, Japan, and Zyla 5.5 camera, Andor Technology, Oxford Instruments, UK) in combination with a 20× objective.

### 4.7 Assessment of Cell Proliferation, Metabolic Activity, and ALP Activity

The total content of cell DNA per sample was quantified using the CyQUANT Cell Proliferation Assay Kit (Thermo Fisher Scientific) following the manufacturer's instructions and used to assess cell proliferation and normalize ALP activity levels. The cells were cultured for 10 days in FC‐Chips and on corresponding 2D controls in GM or DM. Briefly, after 10 days of culture, the medium was removed, and the samples were washed three times with PBS. After three freeze‐thaw cycles (freezing was at −80°C for 30 min followed by incubation at RT for 30 min), lysis buffer from the kit (300 µl) was added to each well and incubated at RT for 1 h. Subsequently, the FC‐Chips were disassembled, and the cells were scraped from both membranes. 50 µl of cell lysate was used for ALP quantification and kept at −30°C until further use. The rest of the lysate was incubated with proteinase‐K solution at 56°C for 18 h, freeze–thawed three times (freezing was at −80°C for 30 min, followed by incubation at RT for 30 min), and incubated with a 1:200 solution of RNAse in lysis buffer at RT for 1 h to degrade cellular RNA. Next, 100 µl of this liquid was incubated with the same volume of CyQUANT GR‐dye in a black microplate at RT for 15 min in the dark. Fluorescence measurements were performed with a CLARIOstar Plus microplate reader (BGM Labtech, Germany) with an excitation wavelength of 485 ± 10 nm and an emission wavelength of 530 ± 10 nm. A standard curve of known cell numbers vs. measured fluorescence intensities was prepared. The number of cells in a 48‐well plate was 5, 10, 20, 30, 40, 50, 60, 70, 80, 90, and 100k cells. The cells were frozen after 6 h in culture. The total cell number per condition was calculated by subtracting from each measurement of a processed cell lysate the fluorescence reading of the dye‐containing pure lysis buffer and converting the fluorescence signal into a cell number with the help of the standard curve. ALP activity was quantified using the CDPStar reagent (Sigma–Aldrich) following the manufacturer's protocol. 10 µL of cell lysate supernatant, as similarly described in the last paragraph, were transferred to a 96‐well plate. 40 µL of CDPStar reagent was added to each well, followed by 30 min incubation at RT in the dark. Luminescence intensity was read with a microplate reader. The results were normalized to the cell number.

### 4.8 Immunocytochemistry and Fluorescence Imaging

Immunocytochemistry was performed to assess the cell morphology, and the expression of the focal adhesion protein vinculin, the mechanosensor YAP1, and the nuclear envelope marker lamin A/C. After cell culture, the samples were washed with PBS and fixed with 4% (w/v) paraformaldehyde (Sigma–Aldrich) for 20 min at RT. After fixation, the samples were washed three times with PBS, and the cells were permeabilized with 0.01% (v/v) Triton X‐100 (Acros Organics) for 15 min. The samples were then blocked for 1 h at RT with a 1:100 solution of goat serum (Sigma–Aldrich) in ‘PBT’, which here was PBS plus 0.02% Triton X‐100 and 0.5% bovine serum albumin (BSA), or with 5% BSA. Next, the samples were incubated with a primary anti‐vinculin antibody (Abcam; ab196579; conjugated to Alexa Fluor 568) 1:200 diluted in 5% BSA, a primary anti‐YAP1 antibody (Santa Cruz; SC‐376830; conjugated to Alexa Fluor 488) 1:200 diluted in 1% goat serum or a primary anti‐lamin A/C antibody (Abcam; ab185014; conjugated to Alexa Fluor 488) 1:100 diluted in 1% goat serum overnight at 4°C. The samples were washed three times with PBS and then incubated with phalloidin (Thermo Fisher; conjugated to Alexa Fluor 568) 1:100 diluted in PBS for 1 h at RT. After washing again three times with PBS, the nuclei were incubated with Hoechst 33258 (Thermo Fisher) 1:1000 diluted in PBS for 10 min at RT, and another time washed three times with PBS. The samples were then mounted using Fluorescence Mounting Medium (Dako, Agilent) and kept at 4°C until imaging. Stained samples were imaged using fluorescence confocal laser scanning microscopy (TCS SP8, Leica) in combination with a 25X objective for vinculin and YAP1 detection and a 60× objective for lamin A/C detection.

### 4.9 Image Analysis

Cell shape was analyzed through CellProfiler (https://cellprofiler.org/; version 3.1.869) employing a custom‐made pipeline [71]. Briefly, after correction of illumination of the channels, the nuclei were identified as primary objects by global minimum cross‐entropy thresholding from the Hoechst image channel. Subsequently, the cell shape was determined as a secondary object by propagation, and the global minimum cross‐entropy thresholding from the phalloidin image channel was applied. For both channels, missegmented artifacts were removed by adapting the threshold for nucleus and cell size.

The length of vinculin‐containing focal adhesions was calculated using the “Particle Analyzer” function in ImageJ (https://imagej.net/ij/). The corresponding calculation was performed by converting the image into an 8‐bit rendering, and the threshold was set outside the 95%‐pixel intensity distribution to help eliminate background signal.

For lamin A/C quantification, a custom‐made CellProfiler pipeline was used. Briefly, the nucleus morphology was identified using the Otsu adaptive thresholding method on the Hoechst image channel to delineate the nuclear area from the cytoplasmic region of the cell. From the nucleus channel, the overlapping lamin A/C signal was identified, and mean intensity values were quantified.

Perinuclear actin distribution was quantified as previously described using a Fiji (https://fiji.sc/) macro [72]. Briefly, a line was drawn through the cell perpendicular to the long axis of the cell and through the center of the nucleus, and the intensity over that line was measured.

YAP1 expression intensity within cells was quantified by dividing the average pixel intensity of the segmented nucleus by the average pixel intensity of the segmented cytoplasm. For this, from the cells, fluorescence confocal microscopy images were taken with a 1 µm step size to cover the complete height of the cells and then combined in a stack using Fiji to apply its maximum intensity projection module.

### 4.10 Statistical Analysis

The quantitative data presented in this study are based on 3 ≤ N ≤ 7 independent samples. All statistical analysis was performed using Prism (GraphPad, USA; version 9.0). One‐way ANOVA (analysis of variance) followed by Tukey's post‐hoc test was applied to determine the statistical significance for the results of the experiments visualized in Figures 4B,C and 5B and 7B–D and 8 and Figures S1B,C and S6B and S7B and S9, and Student's t‐test for Figure 6B. Significance differences between mean values were assumed for p < 0.05. For data assessed per single cell, for each independent sample, we analyzed and averaged n = 30–70 cells. Before applying ANOVA to the averaged sample values, the normality or Gaussian distribution of the data was assessed using the Shapiro‐Wilk test.

## Author Contributions

U.T. and F.G. performed formal analysis, investigation, methodology, validation, visualization, writing – original draft, writing – review & editing. E.G.K. performed investigation, writing – review & editing. T.D.B. performed investigation, writing – review & editing. L.M. performed supervision, writing – review & editing. Z.T.B. performed methodology, supervision, validation, writing – review & editing. C.V.B. performed funding acquisition, writing – review & editing. P.H. performed funding acquisition, methodology, supervision, validation, writing – review & editing. S.G. performed funding acquisition, methodology, supervision, validation, writing – review & editing. J.D.B. performed funding acquisition, methodology, supervision, validation, writing – review & editing. R.T. performed conceptualization, funding acquisition, methodology, supervision, validation, writing – review & editing.

## Funding

Dutch province of Limburg, nos. SAS‐2014‐00837 and SAS‐2018‐02477; Netherlands Organization for Scientific Research (Nederlandse Organisatie voor Wetenschappelijk Onderzoek; NWO), nos. 024.003.013 and 18748; Study Abroad Program of the Turkish Ministry of National Education.

## Conflicts of Interest

R.T. and S.G. are founders, shareholders, and managing directors of the company 300MICRONS GmbH, which is active in the field of 3D cell culture solutions.

## Supporting information




**Supporting File**: adhm71000‐sup‐0001‐SuppMat.docx.

## Data Availability

The data that support the findings of this study are available from the corresponding author upon reasonable request.
