# Supplementary material for: The Floor‐Ceiling‐Chip, or 2 × 2D = Pseudo‐3D—Approaching 3D Cell Morphology and Organization between Two Opposing 2D Substrates with Cell‐Adhesive Protein Micropatterns
Source: Adv Healthc Mater. 2026 Mar 17;15(19):e03591. doi: 10.1002/adhm.202503591 (PMC13206373; doi:10.1002/adhm.202503591)
Supplement: Supplementary file 1 — Supporting File: adhm71000‐sup‐0001‐SuppMat.docx. [file ADHM-15-0-s001.docx]

Supporting Information

The Floor-Ceiling-Chip, or 2 × 2D = Pseudo-3D – Approaching 3D Cell Morphology and Organization Between Two Opposing 2D Substrates with Cell-Adhesive Protein Micropatterns

Urandelger Tuvshindorj, Francesca Giacomini, Esra Güben Kaçmaz, Tim ten Brink, Lorenzo Moroni, Zeinab Tahmasebi Birgani, Clemens van Blitterswijk, Pamela Habibović, Stefan Giselbrecht, Jan de Boer, and Roman Truckenmüller


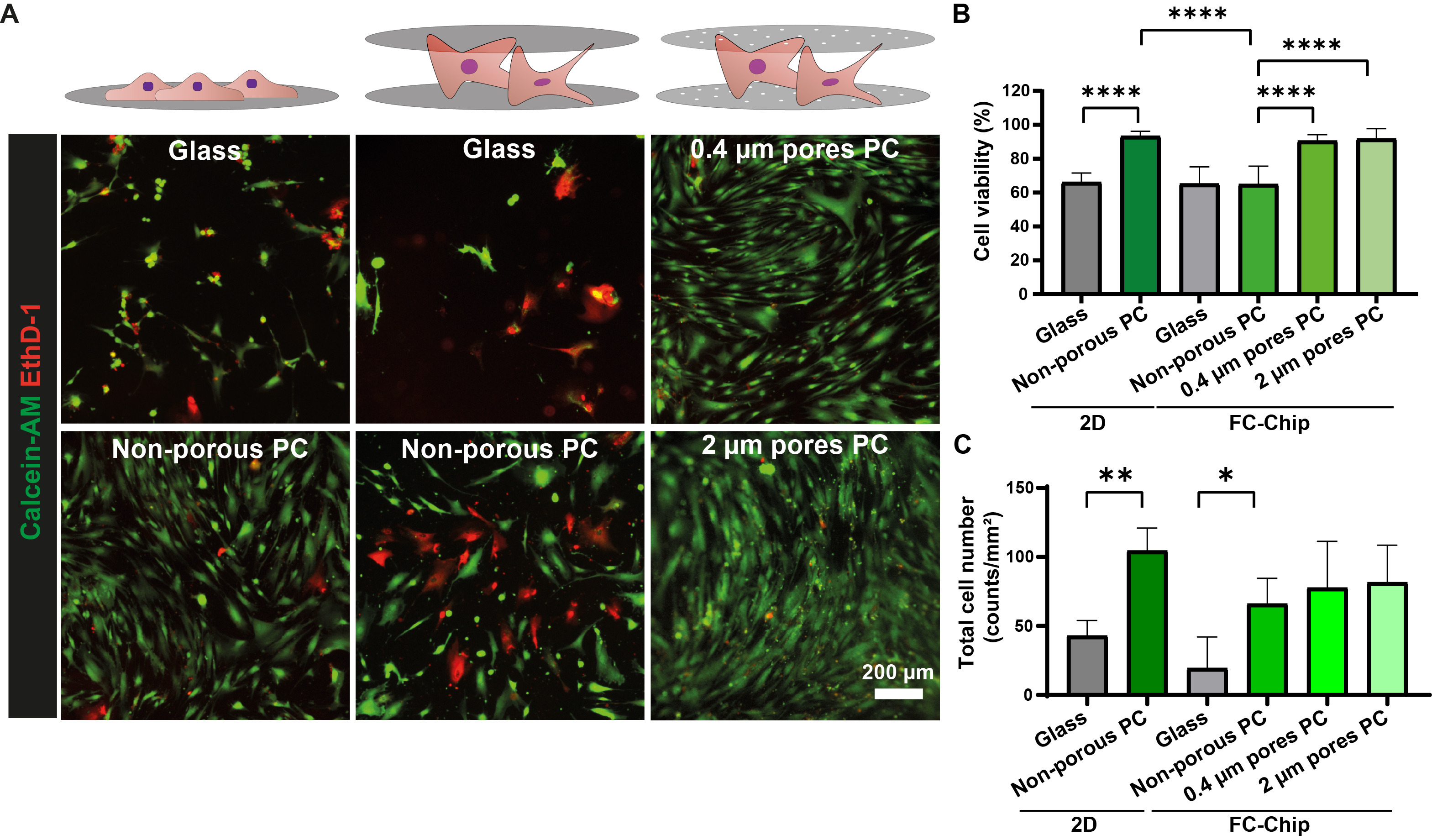


**Figure S1.** Optimization of the FC-Chip (material) design. (A) Fluorescence microscopy images of hMSCs after 10 days of culture on 2D glass or non-porous PC substrates (first column) and in a FC-Chip assembled from the same substrates (second column) or from porous PC membranes with 0.4- or 2-µm diameter pores (third column). Living cells are visualized in green (Calcein-AM) and the dead cells in red (EthD-1). The scale bar applies to all images of the subfigure. (B) Quantification of cell viability, measured as the ratio of Calcein-AM positive, living cells to the total number of (living and dead) cells adhering to the substrates at day 10 of culture. Bars represent mean values and error bars standard deviations. Significance was determined by one-way ANOVA followed by Tukey’s post-hoc test. **** *p* < 0.001. N = 3. (C) Quantification of the total number of cells attached to the substrates after 10 days of culture. Bars represent mean values and error bars standard deviations. Significance was determined by one-way ANOVA followed by Tukey’s post-hoc test. * *p* < 0.05 and ** *p* < 0.01. N = 3.


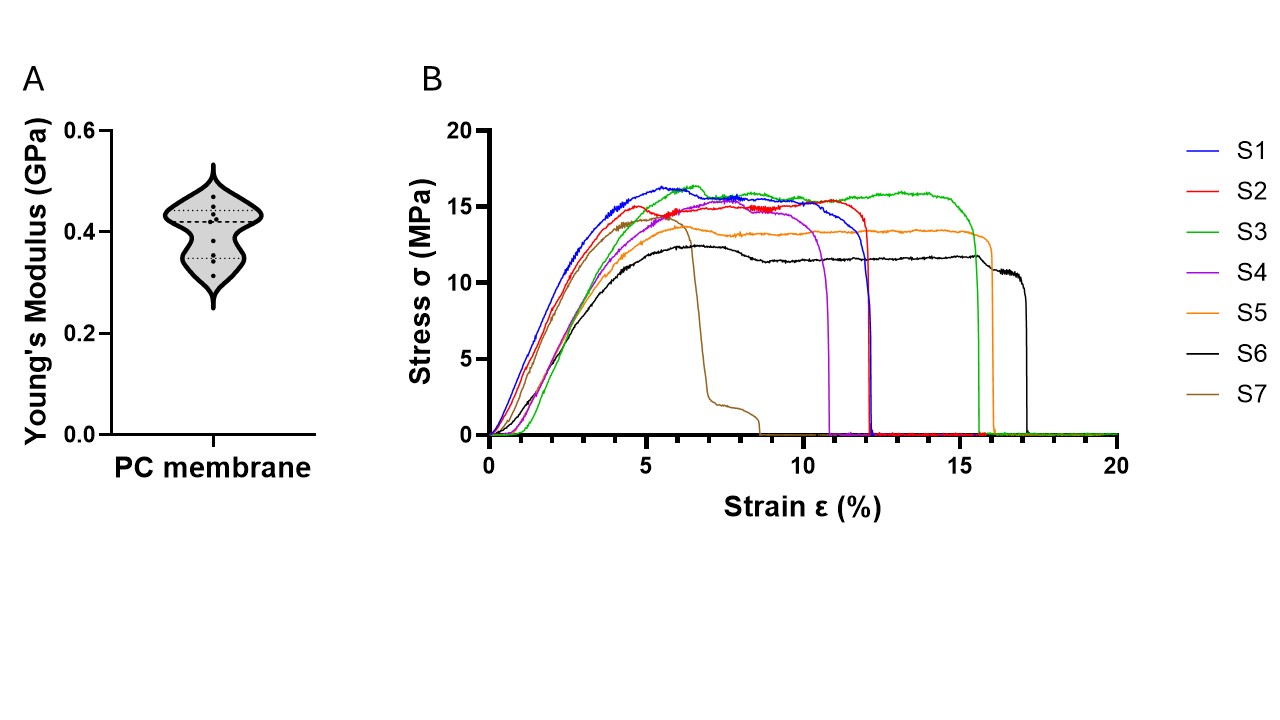


**Figure S2.** Tensile testing of ion track-etched PC membranes. (A) Young’s modulus of membrane determined from tensile testing data. The thicker dotted horizontal line and the two thinner ones represent the median value and interquartile ranges, respectively. N = 7. (B) Stress-strain curve of seven membrane samples S1–S7.


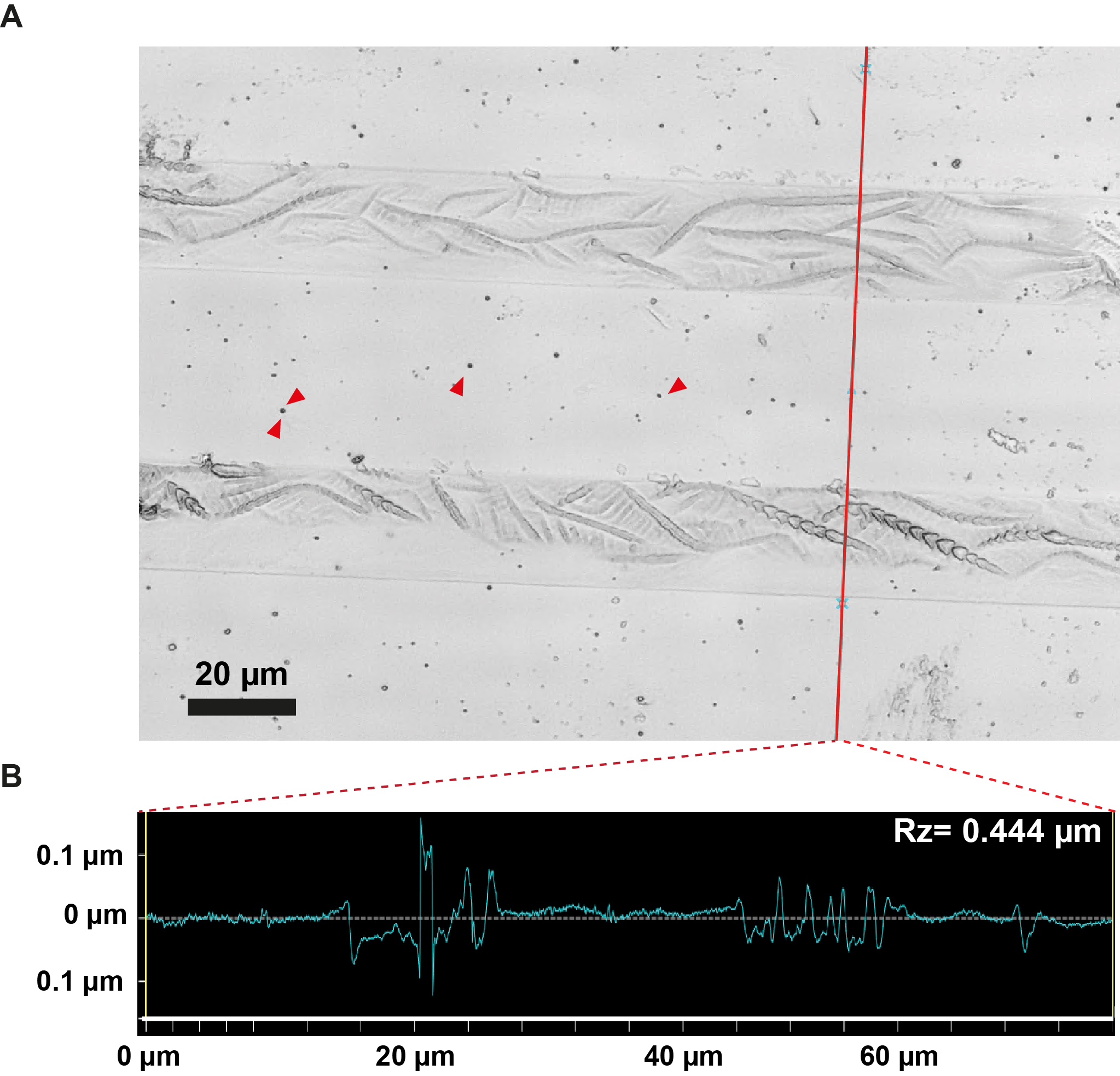


**Figure S3.** Collagen fibril formation in micropatterned regions. (A) Confocal laser scanning microscopy image of a porous PC membrane with a collagen micro line pattern, showing the formation of collagen fibrils in the patterned regions. The red line represents the path taken for measuring the height profile presented in (B). The red arrows indicate the pores of the membrane (0.4 µm diameter, 10^6^ cm^-2^ density). (B) Height profile of the collagen patterns measured using an optical profilometer.


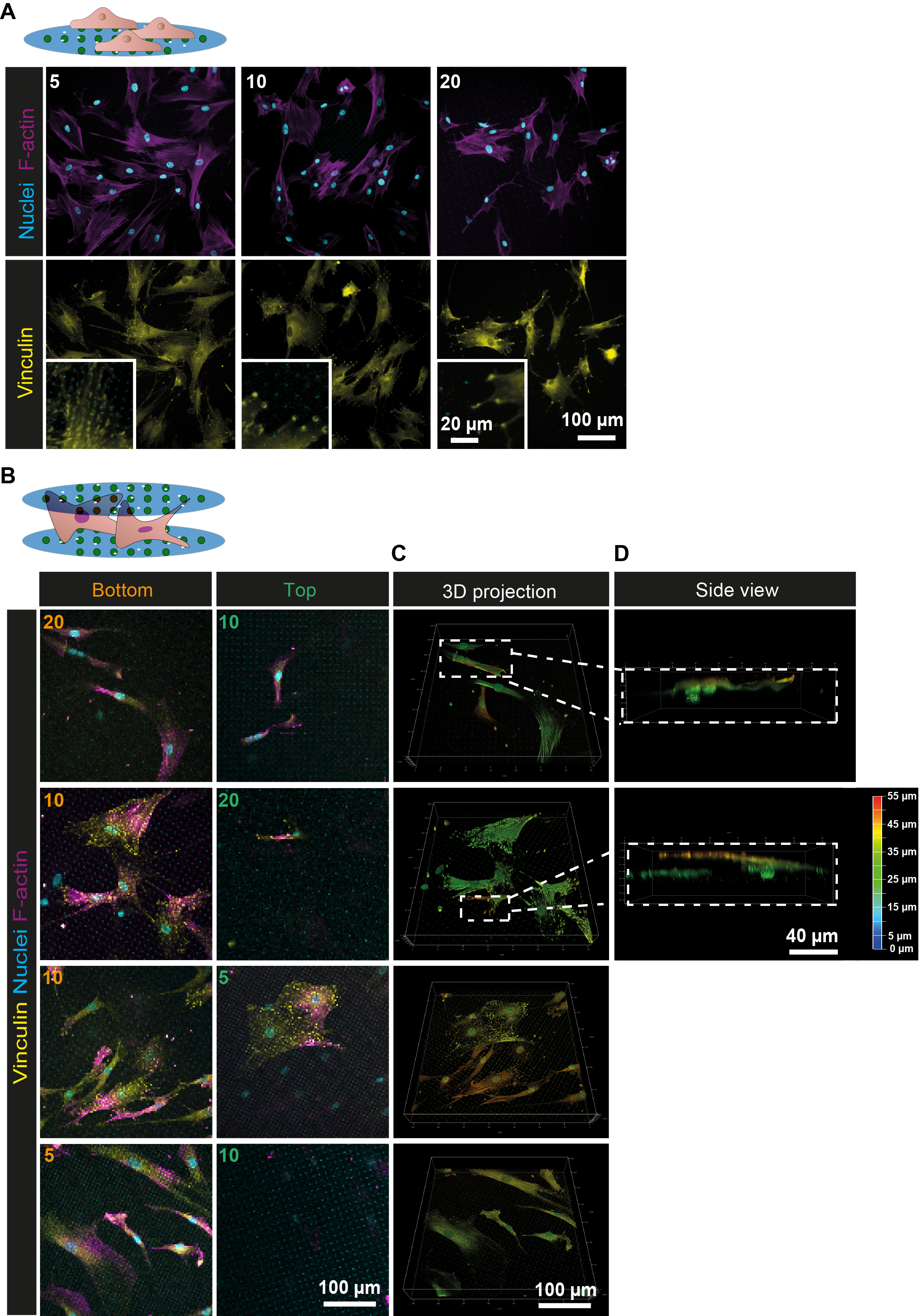


**Figure S4.** Culture in the FC-Chip assembled from combinations of membranes with dot-shaped micropatterns and on corresponding 2D controls. (A) Fluorescence confocal microscopy images of hMSCs cultured for 24 h on 2D controls from porous PC membranes with collagen dot micropatterns with a diameter of 3 µm and a spacing between them of 5 (left), 10 (middle), or 20 µm (right). The cells were stained for vinculin (yellow), F-actin (magenta), and nuclei (blue). The scale bars in the regular images and zoomed-in insets apply to all similar images of the subfigure. (B) Fluorescence confocal microscopy images of hMSCs cultured for 24 h on FC-Chips assembled from the same substrates as used for (A) in the following combinations: in each case on the bottom and top membranes, a spacing of 20 and 10 µm (first row), 10 and 20 µm (second row), 10 and 5 µm (third row), and 5 and 10 µm (fourth row), respectively. The cells were stained for vinculin (yellow), F-actin (purple), and nuclei (blue). The scale bar applies to all images of the subfigure. (C) Fluorescence confocal microscopy image-based 3D reconstruction of cells cultured on the different configurations of the FC-Chip. The scale bar applies to all images of the subfigure. (D) Zoomed-in side-view of cells as shown in (C). The scale bar applies to both images of the subfigure. The color legend indicates the height level and applies to both images of the subfigure.


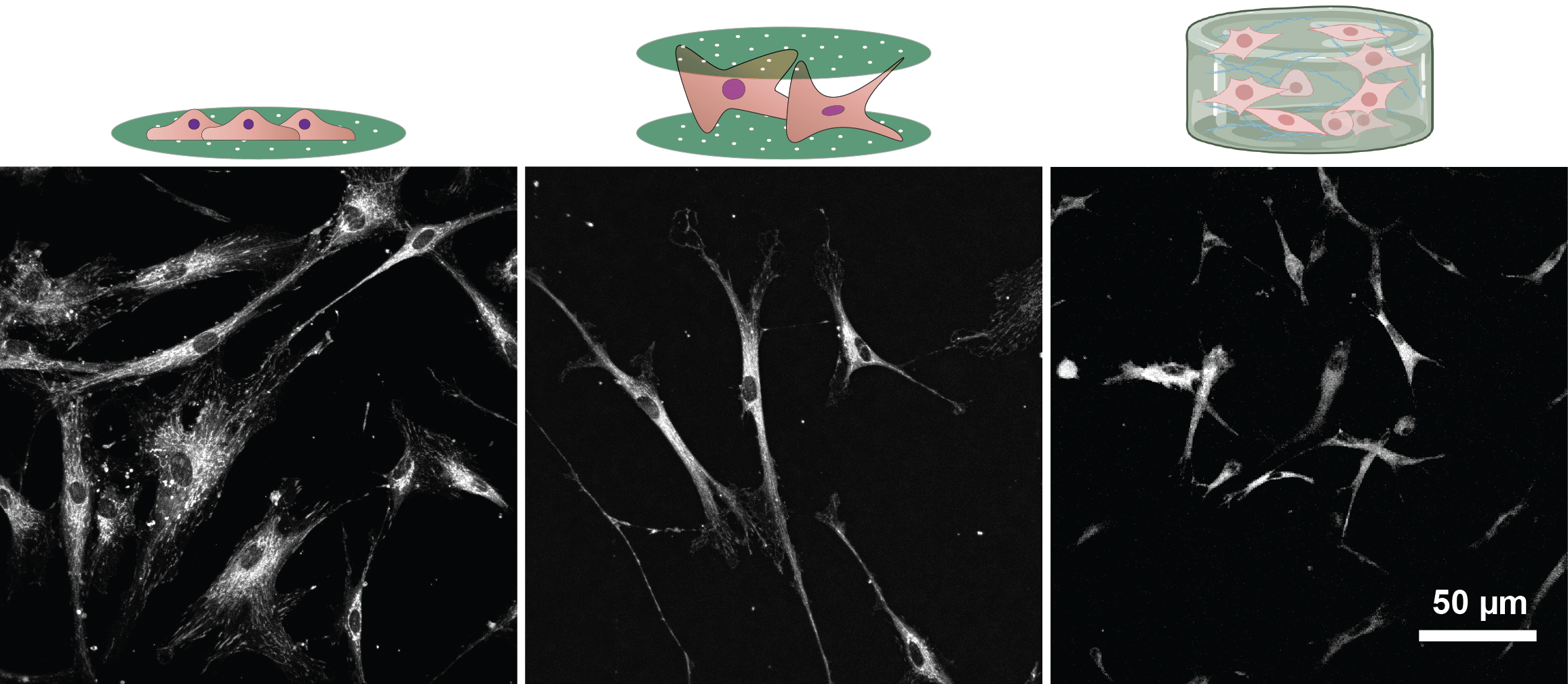


**Figure S5.** Vinculin staining of hMSCs in 2D, FC-Chip, and 3D collagen gel cultures. Fluorescence microscopy images of hMSCs cultured for 24 h on uniformly collagen-coated 2D substrates (first column), in FC-Chips assembled from the same substrates (second column), and in 3D collagen gels (third column). The cells were stained for vinculin (grey). The scale bar applies to all images of the figure.


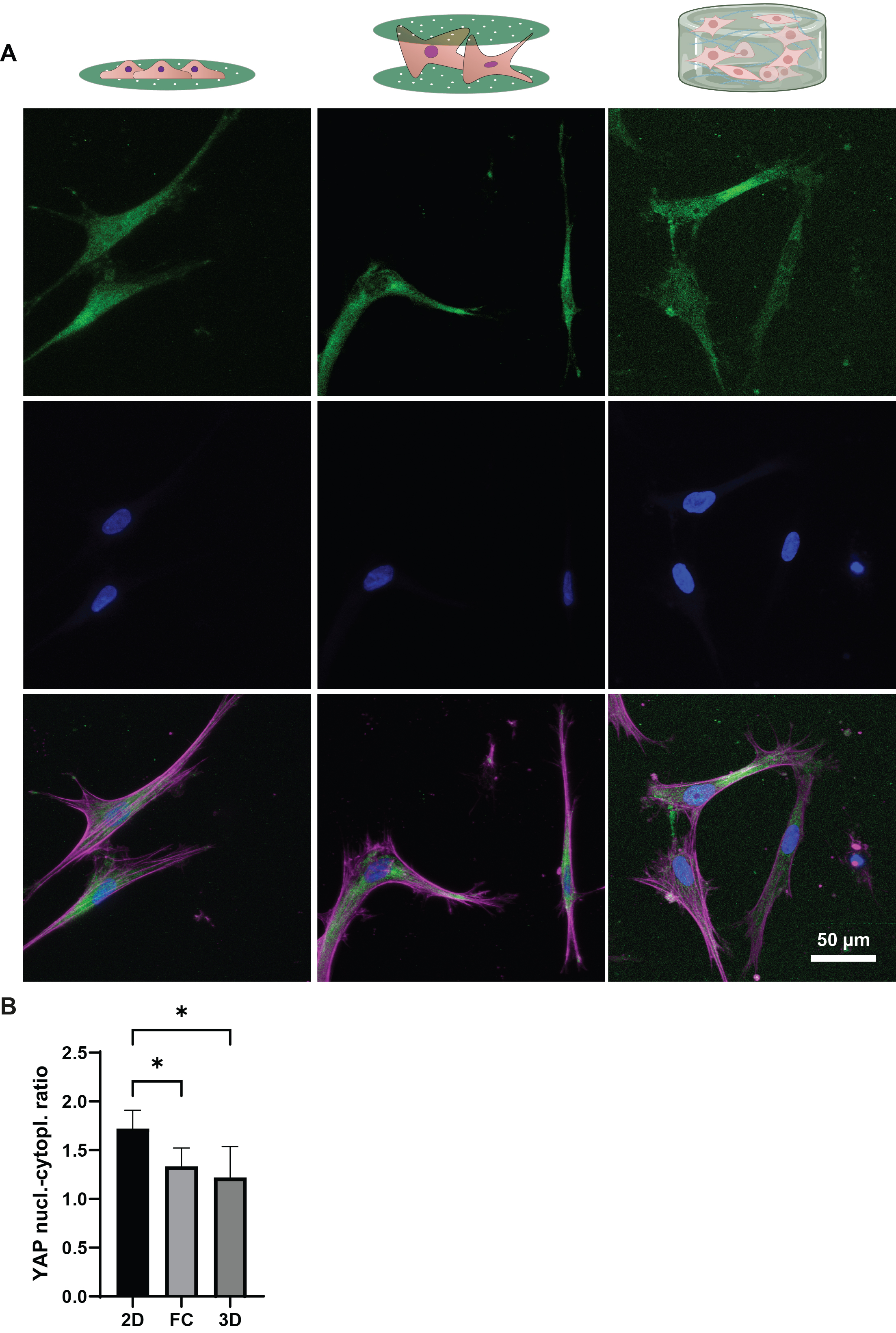


**Figure S6.** YAP translocation in hMSCs in 2D, FC-Chip, and 3D collagen gel cultures. (A) Fluorescence confocal microscopy images of hMSCs cultured for 24 h on uniformly collagen-coated 2D substrates (first column), in FC-Chips assembled from the same substrates (second column), and in 3D collagen gels (third column). The cells were stained for YAP1 (green), F-actin (purple), and nuclei (blue). The scale bar applies to all images of the subfigure. (B) Quantification of YAP1 nuclear-cytoplasmic ratios in 2D, FC-Chip, and collagen gel culture conditions. Bars represent mean values and error bars standard deviations. Significance was determined by one-way ANOVA followed by Tukey’s post-hoc test. * *p* < 0.05. N = 3.


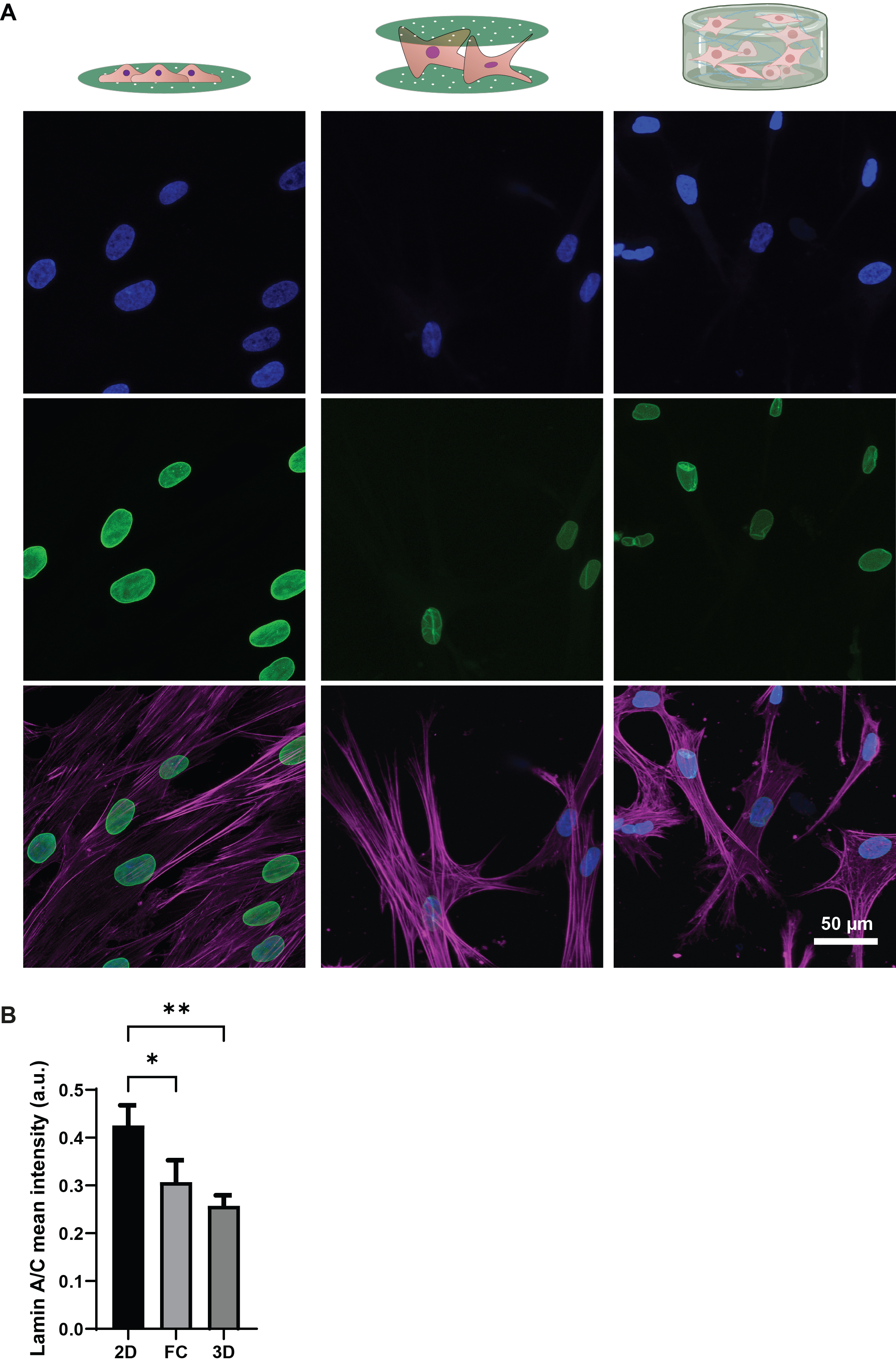


**Figure S7.** Lamin A/C expression of hMSCs in 2D, FC-Chip, and 3D collagen gel cultures. (A) Fluorescence confocal microscopy images of hMSCs cultured for 24 h on uniformly collagen-coated 2D substrates (first column), in FC-Chips assembled from the same substrates (second column), and in 3D collagen gels (third column). The cells were stained for lamin A/C (green), F-actin (purple), and nuclei (blue). The scale bar applies to all images of the subfigure. (B) Quantification of the mean fluorescence intensity of lamin A/C in 2D, FC-Chip, and collagen gel culture conditions. Bars represent mean values and error bars standard deviations. Significance was determined by one-way ANOVA followed by Tukey’s post-hoc test. * *p* < 0.05 and ** *p* < 0.01. a.u. … arbitrary units. N = 3.


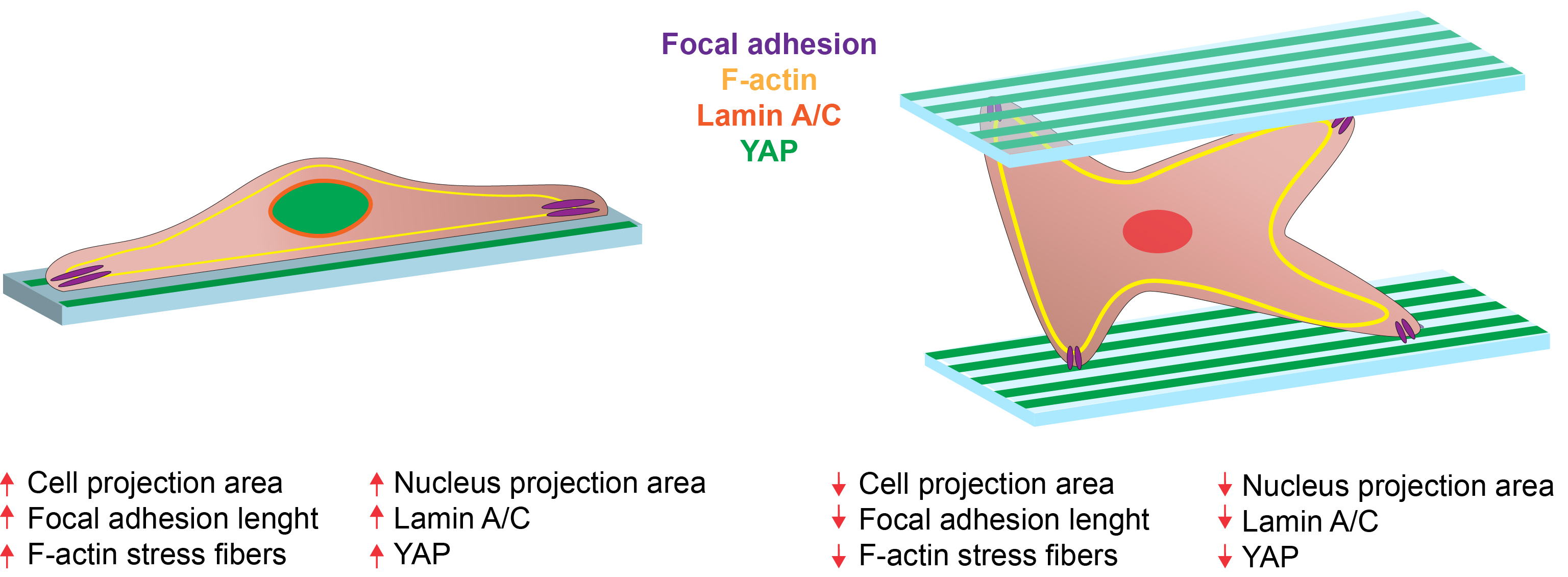


**Figure S8.** Schematic representation illustrating the phenotypic characteristics of hMSCs cultured on 2D substrates (left) compared to FC-Chips (right). Cells cultured on 2D substrates exhibit increased cell area, focal adhesion length, actin stress fibers, and nuclear area, along with elevated expression of lamin A/C and nuclear localization of YAP. In contrast, cells in the FC-Chip, where they are positioned between two opposing substrates, display reduced cell area, shorter focal adhesion length, fewer stress fibers, smaller nuclear area, and lower levels of lamin A/C and nuclear YAP localization. This illustrates how the FC-Chip configuration mitigates some of the artificial polarization and mechanical tension typical of 2D cultures, creating a more physiologically relevant 3D-like environment for the cells. Arrows indicate increase or upregulation and decrease or downregulation between the conditions.


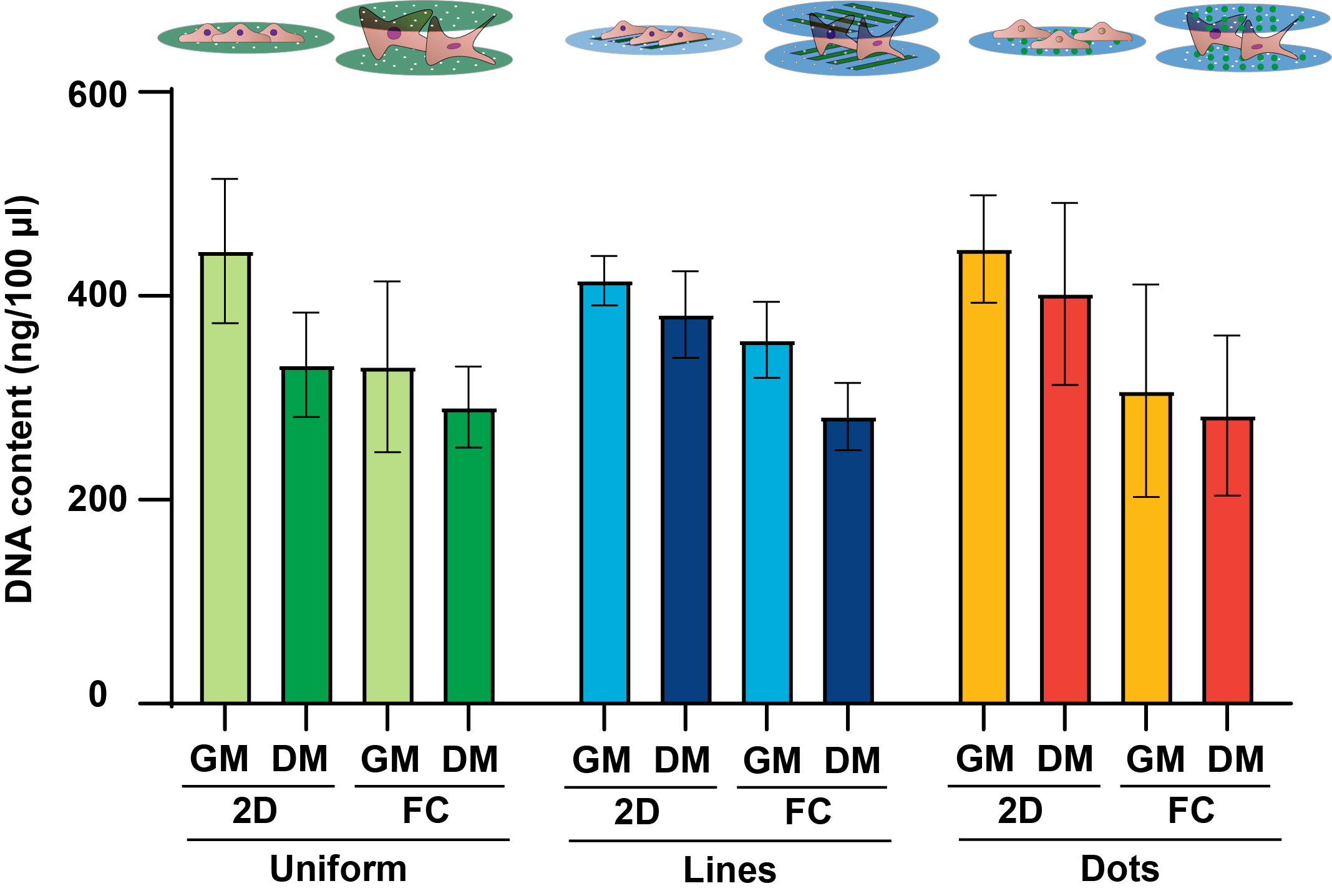


**Figure S9.** Cell proliferation. Quantification of DNA content of hMSCs cultured for 10 days on uniformly collagen-coated and collagen line- and dot-patterned 2D controls, the latter with 10 um spacing, and in corresponding FC-Chips in GM and DM. Bars represent mean values and error bars standard deviations. Significance was determined by one-way ANOVA followed by Tukey’s post-hoc test. No statistically significant differences were found between the groups. N = 3. GM … growth medium; DM … differentiation medium.

**Table S1.** Overview of different collagen patterns used in the study.

| Micropattern name in article | Micropattern feature dimension and spacing | Figure for which micropattern was used |
| --- | --- | --- |
| Squares | 20 µm side length and   - 100 µm spacing | Figures 2B and 3 |
| Lines | 10 µm width and   - 20 µm spacing | Figures 2B, C, and E, 4, 5, 7, 8, S3, and S9 |
| Dots | 3 µm diameter and   - 5 µm spacing - 10 µm spacing - 20 µm spacing | Figures 2B and S4  Figures 8, S4, and S9  Figures S4 |
